# Epidemiological trends of pyogenic spondylodiscitis in Germany: an EANS Spine Section Study

**DOI:** 10.1038/s41598-023-47341-z

**Published:** 2023-11-18

**Authors:** Andreas Kramer, Santhosh G. Thavarajasingam, Jonathan Neuhoff, Hariharan Subbiah Ponniah, Daniele S. C. Ramsay, Andreas K. Demetriades, Benjamin M. Davies, Ehab Shiban, Florian Ringel

**Affiliations:** 1 Department of Neurosurgery, University Medical Center Mainz, Mainz, Germany; 2 Faculty of Medicine, Imperial College London, London, UK; 3 Department of Academic Neurosurgery, Addenbroke’s Hospital, Cambridge University Hospital NHS Healthcare Trust, Cambridge, UK; 4 Imperial Brain & Spine Initiative, Imperial College London, London, UK; 5 Center for Spinal Surgery and Neurotraumatology, Berufsgenossenschaftliche Unfallklinik Frankfurt am Main, Frankfurt, Germany; 6 Edinburgh Spinal Surgery Outcome Studies Group, Department of Neurosurgery, Royal Infirmary Edinburgh, NHS Lothian, Edinburgh, UK; 7 Department of Neurosurgery, Universitätsklinikum Augsburg, Augsburg, Germany; 8 Spondylodiscitis Study Group, EANS Spine Section, Brussels, Belgium; 9 Department of Neurosurgery, University Medical Center Mainz, Johannes Gutenberg University, Langenbeckstraße. 1, 55131 Mainz, Germany

**Keywords:** Infectious diseases, Epidemiology

## Abstract

Pyogenic spondylodiscitis presents significant diagnostic and therapeutic challenges. In Germany, a comprehensive understanding of its epidemiology and inpatient management outcomes is limited, hindering the optimisation of therapeutic strategies. This study aimed to characterise the evolving epidemiological trends of pyogenic spondylodiscitis in Germany, and concurrently evaluate inpatient management strategies and outcomes. We performed a retrospective population-based study of spondylodiscitis cases in Germany from 2005 to 2021, utilising data from the German Federal Statistical Office database. The parameters assessed were incidence trends, demographic characteristics, inpatient management strategies, and inpatient mortality. The study found a significant rise in the population-adjusted incidence of spondylodiscitis in Germany from 2005 to 2021, increasing by 104% from 5.4 to 11.0 cases per 100,000 individuals (p < 0.001). The highest number of diagnoses was recorded in 2019. Age group-adjusted data revealed the largest relative changes in the “90 + ” age group, followed by the “80–89” and “70–79” age groups. These increases were not solely attributable to population changes but were also confirmed after calculating the age-group-adjusted incidence rates. Additionally, our statistical analysis demonstrated that both age and year significantly influenced the incidence of spondylodiscitis. Over the same period, inpatient mortality also surged significantly by 347% (p < 0.001), with the highest increase recorded in the 90 + age group, observing a 2450% rise (p < 0.001). The mean length of inpatient stay decreased by 15% (p < 0.05). Concurrently, there was a significant increase in surgical interventions using spinal stabilisation procedures (p < 0.001), which might suggest a shift in the treatment paradigm for spondylodiscitis. The results underscore a concerning rise in spondylodiscitis incidence and mortality in Germany, particularly affecting the ageing population. A notable shift towards surgical intervention was observed. The data highlights the urgent necessity for high-level evidence studies comparing surgical versus conservative treatment, thereby guiding optimised therapeutic strategies.

## Introduction

Pyogenic spondylodiscitis is a serious bacterial infection affecting the intervertebral disc space and nearby vertebrae. It has specific types caused by fungi, Mycobacterium tuberculosis, or Brucella. However, the Western world has a higher prevalence of the nonspecific pyogenic type, primarily caused by Staphylococcus aureus, Streptococcus species, Pseudomonas aeruginosa, and Enterococcus species^1–5^.

Patients with pyogenic spondylodiscitis present with a spectrum of clinical manifestations. The severity of the infection, its location in the spine, and the patient’s comorbidities greatly influence these manifestations^2,6,7^. A significant challenge lies in the disease's nonspecific symptomology, often leading clinicians to delay diagnosis.

Timely identification and appropriate intervention are imperative to minimize the associated morbidity and mortality. An in-depth understanding of the disease’s epidemiology is not just academic but pivotal. It provides insights into vulnerable populations, thereby aiding the design of effective preventive strategies. Over the past two decades, a surge in the incidence of spinal infections has been reported^8–12^.

Despite the recent contribution by Lang et al. to the epidemiology of pyogenic spondylodiscitis in Germany^13^, there remains a significant and unaddressed question. While Lang and colleagues shed light on the general epidemiology, our primary aim is to discern: Is this uptick genuine across age groups, or is it merely a reflection of an ageing population with a consistent incidence within specific age cohorts? As we move forward, considering the evolving surgical tools and techniques, it’s imperative to ascertain whether there’s a paradigm shift in management strategies. However, understanding the true nature of the disease's incidence, especially within distinct age groups, remains at the forefront of our investigation.

Therefore, the present study aimed to assess the age-group-specific incidences of non-specific spondylodiscitis in Germany. Furthermore, it seeks to analyse in-hospital mortality trends and the prevalence of instrumented surgical interventions. Data from inpatients treated for pyogenic spondylodiscitis were obtained from the Federal Office of Statistics (Statistisches Bundesamt/Destatis) for this purpose.

## Methods

In order to characterise the incidence of spondylodiscitis in Germany, the hospital-admitted patient care activity reports from the Destatis database between 2005 and 2021 were analysed. The database contains details of all secondary and tertiary care (inpatient) admissions, accident and emergency attendances, and outpatient appointments across German hospitals. The database offers big data which can be mined and analysed for trends and patterns in relevant quantitative and qualitative healthcare parameters such as the epidemiological evolution of particular diseases, as well as the development in discharge types after in-patient stay, and treatment outcomes. The database is openly available to the public and characterises hospital admission activity stratified by diagnostic (ICD-10) code. Furthermore, the information available also includes treatment measures classified according to the official operation and procedure code (OPS). The OPS codes corresponding to certain ICD codes can be obtained from the linked database upon request.

Codes that were extracted include the number of diagnoses of non-specific spinal infections (total and age-stratified), mean length of hospital stay (total and age-stratified), and discharge type (regular, against medical advice, transfer to other hospitals, discharge to rehab/care home/hospice, death) between 2005 and 2021 clustered per year.

It is pertinent to highlight that our dataset captures patients transferred between acute hospitals, thereby offering a comprehensive account of mortalities within these settings. However, patients who were transferred to rehabilitation centres or other secondary healthcare facilities, and might have subsequently passed away there, are not encompassed within our dataset.

Given the absence of a singular ICD-10 code uniquely identifying pyogenic non-specific spondylodiscitis, we determined cases based on three ICD-10 codes, which are conventionally utilized to represent this disease: M46.2 (“Osteomyelitis of vertebra”), M46.3 (“Infection of intervertebral disc (pyogenic)”), and M46.4 (“unspecified discitis”). For the scope of our analysis, we scrutinized cases where the coded OPS codes, specifically 5-836 (“spondylodesis”), 5-837 (“vertebral body replacement”), and 5-838 (“complex reconstructions of the spinal column”), were directly associated with the aforementioned ICD-10 diagnoses. This ensures that we are observing surgical interventions that are explicitly linked to the spondylodiscitis diagnosis.

The incidence was then calculated using absolute mid-year population estimates and the relative distribution of age groups, sourced from Destatis.

### Statistical analysis

Descriptive statistics were used to summarize the data on diagnoses, the type of discharges, and mortality rates (Table S1, Supplementary Material) for spondylodiscitis between 2005 and 2021, overall and per age group. Data cleaning was minimal as the dataset was largely complete, and no imputation was required. To calculate the population-adjusted incidence, the Destatis data variables for each year were divided by the German population mid-year estimate of the respective year. Furthermore, to accurately understand the incidence of the disease amidst the epidemiological transition caused by demographic changes, the incidence was calculated for each age group separately by considering the annual proportional share of each age group in the total population. A trend analysis was performed to determine if there was a significant increase or decrease in the incidence over time. The mortality:total diagnoses ratio was also calculated to determine whether there was a shift in treatment outcomes. To examine the age distribution of patients with spondylodiscitis, the mean age and IQR were calculated. To investigate any discharge type differences in spondylodiscitis incidence, a comparative analysis of deaths versus regular discharge versus discharges against medical advice versus transfer to another hospitals versus transfer to rehab/hospice after in-patient stay for spondylodiscitis was performed. To explore changes in length of stay over time, the mean length of stay was calculated for each year between 2005 and 2021. To analyse the number of deaths and diagnoses, pairwise comparisons by age were made using t-tests with pooled standard deviation, adjusting the p-values using the Bonferroni method. The impact of age and year on spondylodiscitis incidence was assessed using a two-way ANOVA, followed by Tukey's test for post-hoc pairwise comparisons. Both multivariate and univariate linear regressions were carried out, considering outcomes related to spondylodiscitis: number of diagnoses, deaths, and length of stay. These analyses factored in explanatory variables including year, age, incidence, types of discharge, death, transfer to other hospitals, proportion of discharge being death, diagnoses with spinal fusion, vertebral body replacement, and complex spinal reconstruction (Tables S2, S3, S5–S8, Supplementary Material). Statistical analyses and graph syntheses were conducted using the R software (version 4.0.4). A p-value of less than 0.05 was considered statistically significant (p < 0.05).

## Results

In total, the data analysed between 2005 and 2021 revealed 131,982 admissions for pyogenic non-specific spondylodiscitis (ICD-10: M46.4 + M46.3 + M46.2) with 78,881 regular discharges, 1289 discharges against medical advice, 6840 deaths, 2847 transfers to other hospitals and 15,890 discharges to rehab facilities/care home/hospice after in-patient stay for spondylodiscitis.

### Total diagnoses & population-adjusted incidence

**Figure 1:**
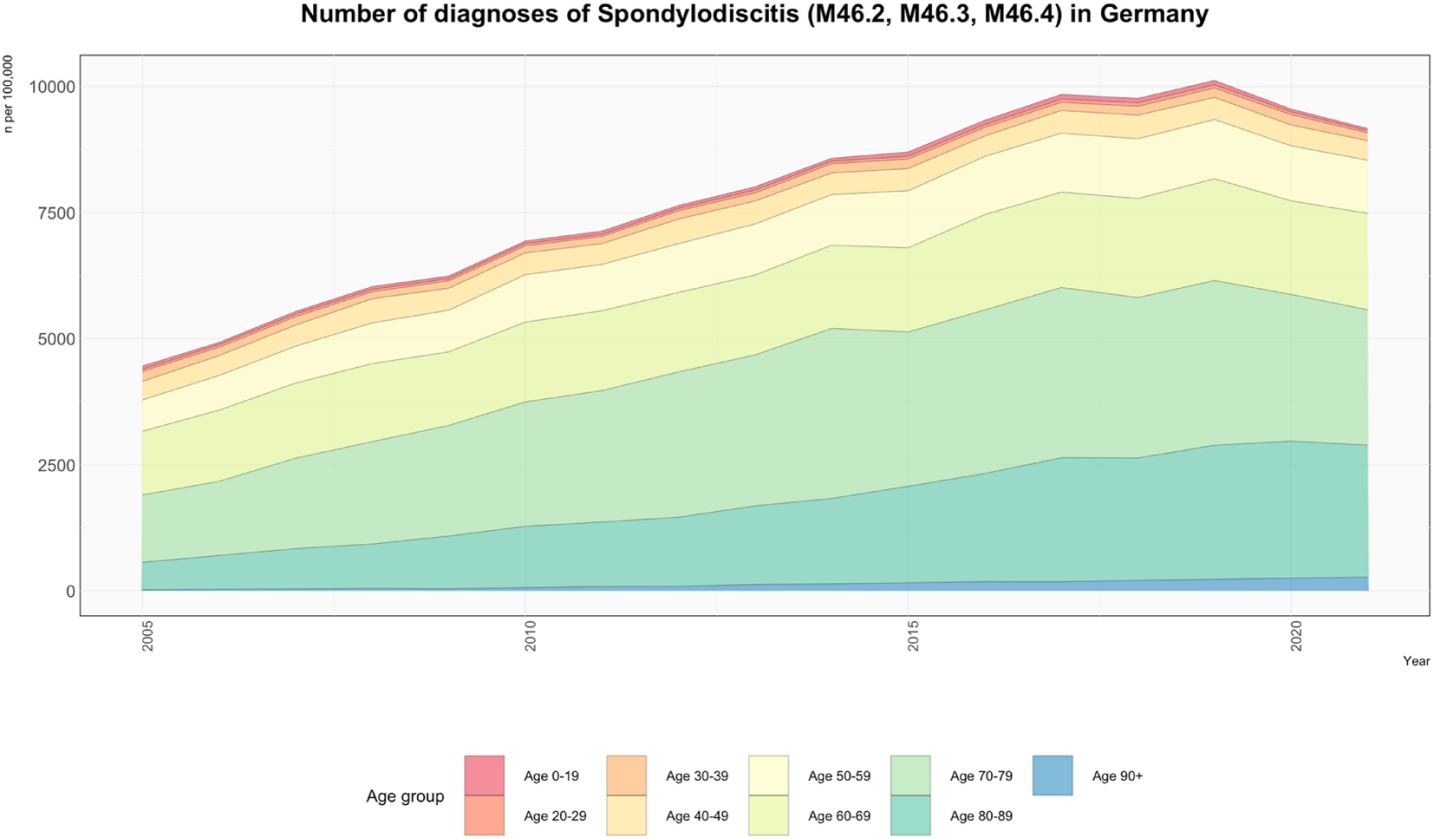
This area chart visually represents the number of spondylodiscitis diagnoses (categorized by ICD-10 codes: M46.2, M46.3, M46.4) in Germany from 2005 to 2021. The distinct color layers correspond to different age groups, illustrating the distribution of these diagnoses across age groups. The vertical axis represents the number of diagnoses per 100,000 of the general German population, while the horizontal axis spans the years under observation.

**Figure 2:**
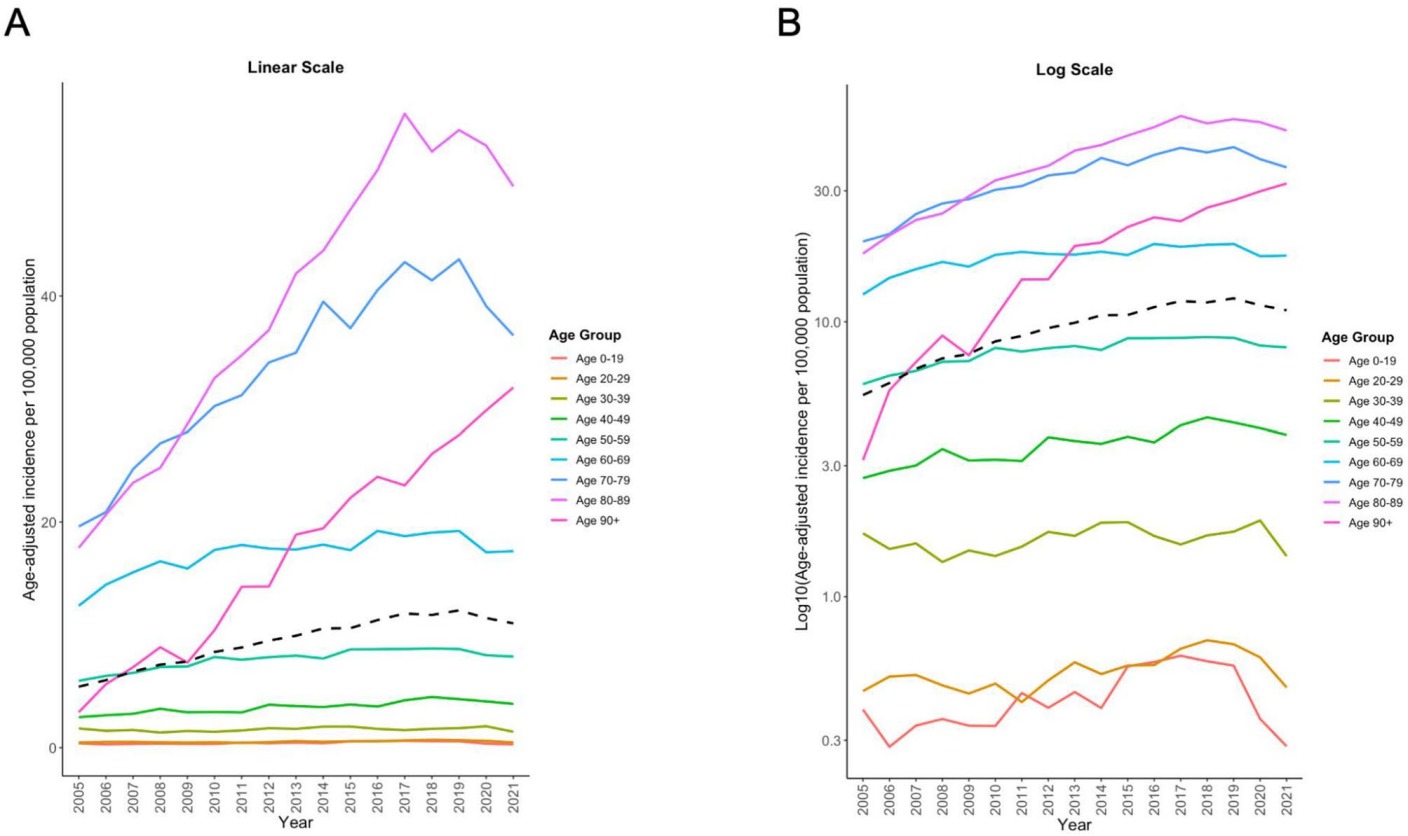
The presented line plots elucidate the age-adjusted incidence rates of spondylodiscitis (ICD-10: M46.2, M46.3, M46.4) in Germany spanning the years 2005 to 2021, distinctly represented for various age groups. Panel (**A**) utilizes a linear scale, making evident the absolute changes over the years, while Panel (**B**) employs a logarithmic scale, emphasizing the rate of change across age categories. The black dashed line in both panels symbolizes the cumulative incidence rate across all age groups, offering a consolidated view of the disease’s prevalence each year.

Diagnoses for spondylodiscitis increased by 106%, from 4457 in 2005 to 9167 in 2021 (Fig. 1). The peak was in 2019, with 10,121. The population-adjusted incidence of spondylodiscitis for all age groups combined from 2005 to 2021 averaged 9.5 diagnoses per 100,000 population. The incidence increased by 104%, from 5.4 in 2005 to 11.0 in 2021 (Fig. 2) with the peak in 2019 at 12.2 diagnoses per 100,000 population (Table S1, Supplementary Material).

### Age group-adjusted number of diagnoses

The 70–79 age group had the most diagnoses with 44,864 between 2005 and 2021 (34% of the total). This was followed by the 60–69 age group (28,344, 21.5%) and the 80–89 age group (27,989, 21.2%). The highest single-year diagnosis for spondylodiscitis in 2021 was for the 70–79 age group with 2686, and the 80–89 age group with 2618 (Fig. 1).

### Age group-adjusted incidence

To characterize the rise in incidence against the epidemiological transition due to demographic change, we assessed the incidences for all age groups, accounting for their annual proportional share of the population.

The top three age groups with significant relative changes from 2005 to 2021 were identified. The “90 + ” age group experienced the most dramatic increase, with an incidence rate that went from 3.14 in 2005 to 31.90 in 2021. This was followed by the “80–89” and “70–79” age groups, with incidence rates of 49.72 and 36.51 in 2021, respectively.

In our investigation, seven age groups demonstrated a rise in spondylodiscitis incidence from 2005 to 2021 that surpassed predictions based solely on demographic growth.

For the 20–29 age group, we noted a modest increase in incidence from 0.45 per 1000 individuals in 2005 to 0.47 in 2021. Adjusted for demographic changes, the expected incidence was 0.44, showing that the observed rise exceeded the predicted figure by 0.03. A similar trend was seen in the 50–59 age group: the observed incidence rose to 8.07 per 1000 individuals in 2021, exceeding the predicted 7.40 by 0.67. This trend was even more evident in the 40–49 and 60–69 cohorts, where the observed incidences surpassed the demographic predictions by 1.89 and 3.69, respectively.

There were significant discrepancies in the senior age groups. In the 70–79 and 80–89 categories, the actual spondylodiscitis cases exceeded the demographic predictions by 15.35 and 19.40, respectively. The most striking was the 90 + group, with an observed incidence of 31.90 per 1000 individuals in 2021, outdoing the demographic prediction by a considerable 27.50.

These results suggest that the rise in spondylodiscitis diagnoses is not solely attributable to population growth but points to an actual increase in the disease’s incidence.

A two-way ANOVA results revealed statistically significant effects of both age (F(9, 144) = 147.107, p < 0.001) and year (F(16, 144) = 5.315, p < 0.001) on the incidence. The comparisons within age groups indicated significant differences between various age groups (p < 0.05), while comparisons among years showed significant differences between multiple years (p < 0.05). These findings suggest that both age and year are important factors influencing the incidence of spondylodiscitis.

### Types of spinal surgery

**Figure 3:**
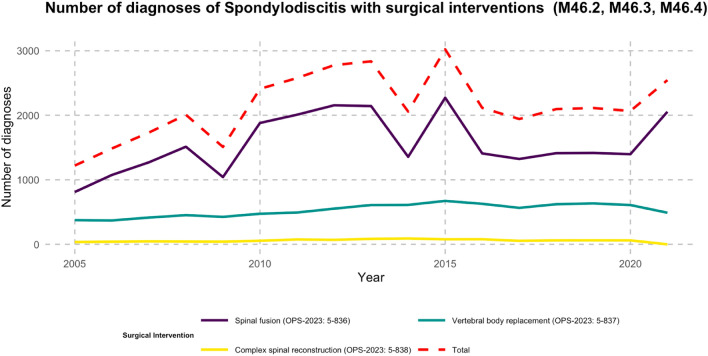
The depicted line chart illustrates the progression of diagnosed spondylodiscitis cases (ICD-10: M46.2, M46.3, M46.4) treated with surgical interventions between 2005 and 2021. The treatments are classified into three specific surgical categories: spinal fusion (OPS-2023: 5-836), vertebral body replacement (OPS-2023: 5-837), and complex spinal reconstruction (OPS-2023: 5-838). A prominent red dashed line signifies the cumulative total of these interventions, providing an encompassing view of the surgical treatment trends over the years.

From 2005 to 2021, spinal instrumented procedures saw a rise of 153.6%. By the end of this period, in 2021, there were a total of 2,675 such procedures (as depicted in Fig. 3). Among all age groups, the most significant surge was observed in the 70–79 age bracket, which experienced a growth of 156.6% in these procedures. Diagnoses related to vertebral body replacement increased by 30.7%. Interestingly, procedures associated with complex spinal reconstruction stopped completely by 2021.

### In-hospital mortality

From 2005 to 2021, there were a total of 6840 in-hospital deaths attributed to spondylodiscitis across all age groups, resulting in an overall in-hospital mortality rate of 5.2%. The year 2021 had the most deaths in a single year with 684, and within that year, the 80–89 age group registered the highest death count with 316 cases.

Between 2005 and 2021, the most striking increase in deaths was seen in the 90 + age bracket: a surge of 2450% or a 25.5-fold rise, moving from just 2 deaths in 2005 to 51 by 2021. Following closely, the 80–89 age group displayed an 829% rise or a 9.29-fold increase, with deaths jumping from 34 in 2005 to 316 in 2021.

Combining all age groups, deaths due to spondylodiscitis soared by 347% or 4.47-fold, going from 153 deaths in 2005 to the aforementioned 684 in 2021. Concurrently, the overall ratio of deaths to diagnoses climbed by 117.4% or a 2.17-fold increase, shifting from 0.034 in 2005 to 0.075 in 2021. In 2021, the 90 + age group had the most pronounced death-to-diagnosis ratio at 0.1889.

### Length of stay

On average, the duration of hospital stays saw a reduction of 15% between 2005 and 2021. The highest length of stay in 2021 was recorded for age 60–69 years with 24.3 days, the second highest length of stay was recorded for age 70–79 years with 24.7 days. The highest decrease in length of stay was for ages 20–29 years (43% decrease, from 18.1 days in 2005 to 10.2 days in 2021). Overall, the trend is decreasing, albeit fluctuating. In regression analyses, Year and Age categories were found to significantly correlate with length of stay (LOS), with nearly all p-values < 0.05 (Table S8, Supplementary Material).

### Types of discharge

**Figure 4:**
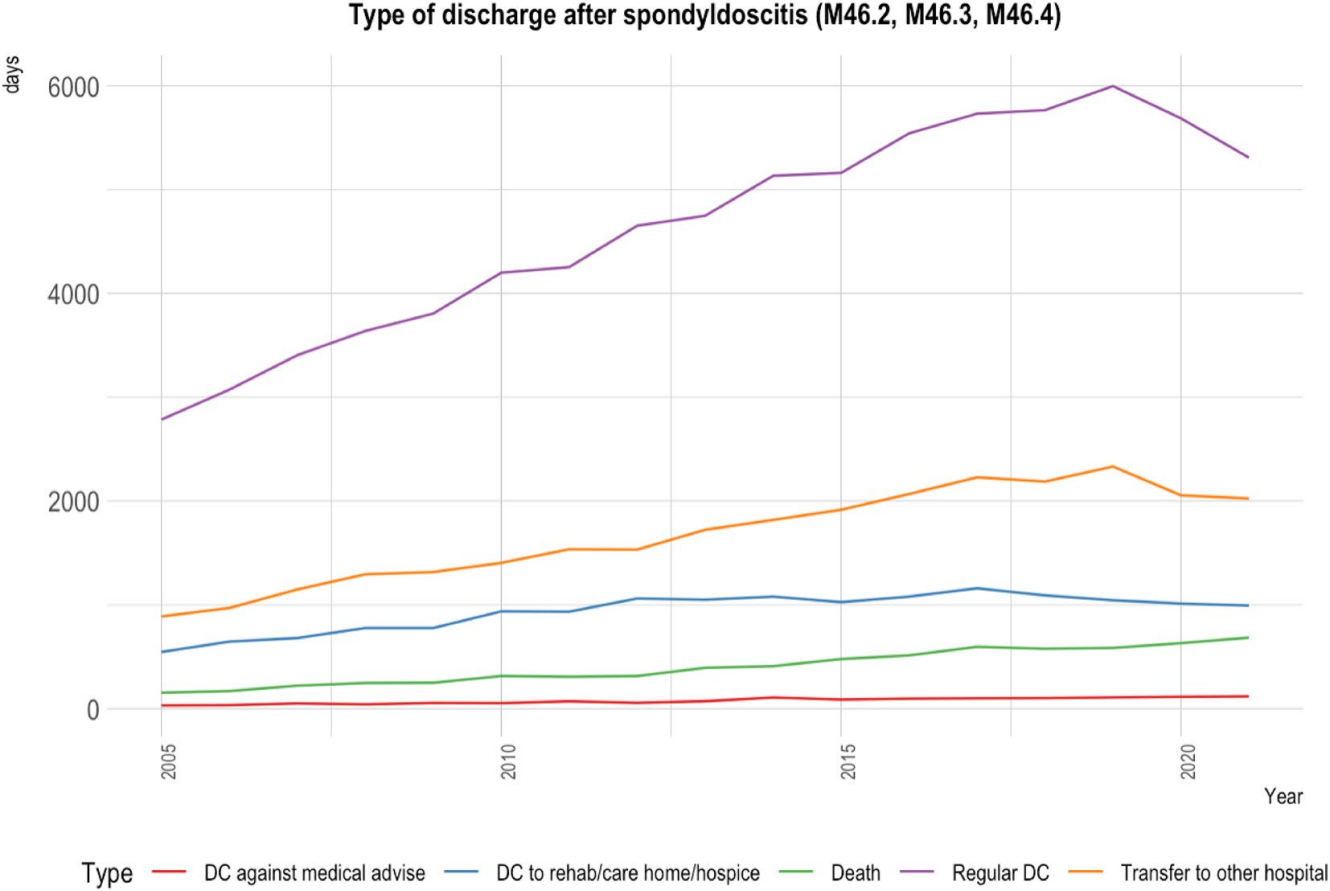
Displayed is a line chart delineating the distinct types of patient discharges after being hospitalised for spondylodiscitis (ICD-10: M46.2, M46.3, M46.4) spanning the years 2005 through 2021. The types of discharges include those discharged against medical advice, transferred to rehabilitation or care homes/hospice, instances of death, regular discharges, and transfers to other medical institutions. This representation offers a comprehensive perspective on patient outcomes and trends for those diagnosed with spondylodiscitis over the 16-year period.

Changes in discharge methods were evident between 2005 and 2021, as shown in Fig. 4. Regular discharges rose by 90% (from 2785 to 5308) (Table S1, Supplementary Material). Meanwhile, patients discharged against medical advice saw an increase of 293% from 30 for all age groups combined in 2005, to 118 (Table S1, Supplementary Material). Notably, the number of deaths was negatively correlated with the number of discharges against medical advice (p < 0.05) (Table S7, Supplementary Material). Transfers to other medical facilities went up by 127.9%, and transfers to rehabilitation centres, care homes, or hospices increased by 81.9%.

## Discussion

The primary objective of the present study was to illustrate the evolution of pyogenic spondylodiscitis incidence and spotlight its inpatient treatment modalities and associated outcomes within Germany, utilising the reporting data of the Federal Statistical Office.

### Epidemiological trends and demographic implications

The population-adjusted analysis indicated a near-linear growth in incidence during the investigation period, almost doubling to 11.0 per 100,000 from 2005 to 2021. The peak incidence was noted in 2019 at 12.2 per 100,000, the highest ever recorded within Germany. Previous literature has documented a surge in incidence but to a more conservative extent, between 0.2 and 5.8 cases per 100,000 per annum^14–16^. Our research group recently reported similar elevated figures in England (11.6 per 100,000 population in 2019/2020)^12^, with comparable statistics reported in France (peak incidence of 11.3 per 100,000 in 2019)^11^. However, the current data does not clarify whether the rise in cases is due to the ageing population or an actual increase in the disease’s incidence alongside rising comorbidities and immunosuppressive treatments. To investigate this, we considered the annual proportional share and its changes over time for each age group when calculating the incidence of spondylodiscitis. This helps determine if the increase in diagnoses reflects a genuine rise in incidence or is a result of the shifting demographics of an ageing society.

When analyzing age group-adjusted incidence, three age groups stood out with significant relative changes between 2005 and 2021. The age group “90 + ” exhibited the highest relative change of approximately 915.13%, followed by the “80–89” and “70–79” age groups. Among others, these age groups also showed an incidence increase that exceeded the growth attributable solely to expectable demographic changes in their respective population size. These changes may encompass advancements in diagnostic capabilities, shifts in healthcare accessibility, changes in comorbidity prevalence, or even alterations in microbial environments. Our findings thus unravel a new narrative around spondylodiscitis epidemiology, suggesting that factors beyond demographic change are driving the condition's rising incidence, yet especially affecting the geriatric population.

### Immune decline and increasing risk factors

The heightened susceptibility to infections within the elderly demographic might be attributed to the gradual decline of the immune system, a process known as immunosenescence^17^. The increased prevalence of frailty with age also contributes to this trend, which in turn is associated with increased disability, hospitalisation, negative health outcomes and overall mortality^18,19^.

These circumstances are paralleled by a marked increase in the typical risk factors for spondylodiscitis, such as diabetes mellitus. The incidence of male patients diagnosed with diabetes in Germany has grown notably fast since 2000, having more than doubled from approximately 7.3 million in 2000 to 16.7 million in 2019. Also, the incidence of women with diabetes in Germany has increased markedly, from 9.5 million in 2000 to 15.6 million in 2019, a rise of more than 50%^20^. It can also be assumed that there is an increase in the prevalence of immunosuppressed patients due to the changing population structure although accurate data on this is not available^21^.

### Projections in surgical interventions

Adding to the weight of these findings, a recent paper Heck et al. provided projections from surgical use models in Germany, indicating a significant rise in spinal fusions, especially in patients 75 years and older^22^. The paper highlighted that the incidence rate of posterior spinal fusion is projected to increase by approximately 83% to 102% per 100,000 inhabitants in 2060. Furthermore, the highest increase was identified in patients aged 75 years and older. With these projections in mind, as the global geriatric population continues to grow, both absolutely and proportionally, the burden on healthcare systems is set to intensify^23,24^.

This increase in spinal surgeries, particularly in the geriatric population, holds multifaceted implications. Aging, with its associated decline in bone density and the presence of pre-existing comorbid conditions, as mentioned above, can present challenges to surgical interventions, ultimately increasing the risk of complications and prolonging recovery. Additionally, the psychological and emotional toll on elderly patients undergoing invasive procedures can be significant, potentially impacting their overall well-being and rehabilitation prospects^25^.

### Mortality trends across age groups

We observed a pronounced increase in mortality, both in terms of absolute numbers and when evaluated relative to reported diagnoses, across all age groups studied. The age groups 80–89 years and 90 + years, in particular, showed marked surges in mortality from 2005 to 2021. The age group 80–89 years reported the highest mortality count in 2021, demonstrating a 9.29-fold escalation since 2005. The age group 90 + exhibited an even more significant upswing, with a 25.5-fold increase over the same period.

In parallel with our in-hospital mortality findings, a Danish nationwide register study highlighted a comparable uptrend in post-hospitalization mortality for spondylodiscitis, emphasizing the role of comorbidities and substance abuse in influencing these outcomes^26^. Such converging data from different geographic regions underscore the gravity of the situation. Although contemporary advancements in diagnostic capabilities and treatment approaches for spondylodiscitis are notable, our data indicates that these developments have not culminated in a substantive reduction in mortality. This suggests potential gaps in either diagnostic acumen, treatment strategies, or post-diagnostic care.

Furthermore, it’s essential to acknowledge that the recorded mortality rates in our dataset might only provide a partial view. It's plausible that the actual threat posed by spondylodiscitis is more formidable than represented, given potential underreporting or the omission of mortality from secondary complications and diseases. Addressing this requires an integrated approach that not only focuses on direct treatment but also encompasses broader preventive and post-care strategies, especially for the vulnerable geriatric population.

### Hospitalisation trends and treatment protocols

Regarding patient hospitalisation, we observed a trend towards a shorter length of stay throughout the study period, although there were occasional fluctuations. Older patients generally required longer inpatient treatment, and the minimum average length of stay remained significantly over two weeks. This can be attributed to a widely implemented standard protocol of two-week intravenous (i.v.) antibiotic therapy, which is an integral component of both conservative and surgical treatment approaches. Detailed guidelines regarding the duration of i.v. antibiotic therapy, which typically necessitates prolonged inpatient care, are currently absent in both German and American spondylodiscitis treatment guidelines^27,28^. The majority of patients returned to their habitual residence post-treatment. However, with the escalating incidence, an increasing number of patients were transferred to other inpatient facilities for further care. Whether early discharge from inpatient treatment is feasible remains undetermined based on existing evidence. The OVIVA study demonstrated that a six-week oral antibiotic regimen was non-inferior to i.v. therapy concerning therapeutic success after one year, albeit the study had a limited number of spondylodiscitis cases^29,30^.

### Evaluation of surgical approaches

Evaluation of reported OPS codes revealed that both the absolute and proportional number of patients undergoing surgical treatment via spinal stabilisation markedly increased during the investigation period. This therapeutic approach was particularly common amongst older patients, possibly contributing to the reduction in hospital stays. However, establishing a causal relationship based on the available data is challenging.

## Conclusion

Our analysis of the Federal Statistical Office data from 2005 to 2021 underscores an alarming uptick in spondylodiscitis incidence in Germany, nearly doubling to 11.0 per 100,000 individuals – and transcending what could be attributed solely to demographic shifts, indicating a genuine escalation of disease incidence. The pronounced susceptibility of the geriatric population merits specific consideration. Concurrently, mortality has been increasing both in absolute terms and relative to reported diagnoses, highlighting the serious clinical impact of this condition.

While our study leverages extensive data from the Federal Statistical Office, it's essential to recognize potential biases due to varying reporting standards across institutions. The introduction of the DRG system in Germany in 2004 could have influenced the early years of our dataset, reflecting an adaptation in hospitals’ coding and reporting. Such variations might lead to potential underrepresentation or misclassification of cases. Although our data captures cases transferred between acute hospitals comprehensively, it misses patients transferred to rehabilitation centres or other secondary healthcare facilities. Consequently, any mortality in those centres remains unaccounted for. The overlapping nature of ICD-10 codes for pyogenic non-specific spondylodiscitis might have inadvertently included some specific spondylodiscitis cases. We acknowledge that over the years, diagnostic standards for spondylodiscitis have evolved, and the accessibility of advanced diagnostic tools such as MRI has improved. These advancements might contribute to the perceived increase in the incidence of the disease due to enhanced detection capabilities. Other confounding factors, like regional healthcare disparities or demographic shifts, could also influence our results.

The observed rise in surgical interventions via spinal stabilisation is noteworthy, but the relationship between treatment modalities and observed mortality rates remains ambiguous given our dataset’s constraints. The trends indicate an impending significant burden on healthcare systems, emphasizing an urgent need for further research. This research must be comprehensive, considering the highlighted limitations, and focus on devising targeted interventions for the most vulnerable subpopulations to counteract this threat.

### Supplementary Information

Supplementary Tables.

## Data Availability

All relevant data supporting the findings of this study can be accessed within the Supplementary Digital Content attached to the article. Additionally, a comprehensive dataset focusing on the epidemiological trends of pyogenic spondylodiscitis in Germany is freely available for the public. This dataset, used for this EANS Spine Section Study, can be retrieved from our dedicated GitHub repository. For ensuring transparency, replicability, and rigorousness of the research, this repository encompasses both the raw and processed data integral to our study. Access the dataset via the following link: https://github.com/kramerneurosurgery/PyogenicSpondylodiscitisTrendsGermany.git. Access the complete R code used in this study via the following link: https://github.com/santhoshgthava/PyogenicSpondylodiscitisTrendsGerman. We strongly encourage fellow researchers and individuals with vested interests to employ these resources in their respective research endeavours and subsequent analyses.

## Abbreviations

ICD: International statistical classification of diseases and related health problems
OPS: Operation and procedure codes (“Operation- und Prozeduren-Schlüssel”)

## References

[1] Hadjipavlou AG, Mader JT, Necessary JT, Muffoletto AJ (2000). Hematogenous pyogenic spinal infections and their surgical management. Spine.

[2] Krogsgaard MR, Wagn P, Bengtsson J (1998). Epidemiology of acute vertebral osteomyelitis in Denmark: 137 cases in Denmark 1978–1982, compared to cases reported to the National Patient Register 1991–1993. Acta Orthop. Scand..

[3] Malawski SK, Lukawski S (1991). Pyogenic infection of the spine. Clin. Orthop. Relat. Res..

[4] Grammatico L, Baron S, Rusch E, Lepage B, Surer N, Desenclos JC (2008). Epidemiology of vertebral osteomyelitis (VO) in France: Analysis of hospital-discharge data 2002–2003. Epidemiol. Infect..

[5] Mylona E, Samarkos M, Kakalou E, Fanourgiakis P, Skoutelis A (2009). Pyogenic vertebral osteomyelitis: A systematic review of clinical characteristics. Semin. Arthritis Rheum..

[6] Rezai AR, Woo HH, Errico TJ, Cooper PR (1999). Contemporary management of spinal osteomyelitis. Neurosurgery.

[7] Weinstein MA, Eismont FJ (2005). Infections of the spine in patients with human immunodeficiency virus. J. Bone Jt. Surg. Am..

[8] Jensen AG, Espersen F, Skinhoj P, Frimodt-Moller N (1998). Bacteremic *Staphylococcus*
*aureus* spondylitis. Arch. Intern. Med..

[9] Jensen AG, Espersen F, Skinhoj P, Rosdahl VT, Frimodt-Moller N (1997). Increasing frequency of vertebral osteomyelitis following *Staphylococcus*
*aureus* bacteraemia in Denmark 1980–1990. J. Infect..

[10] Musher DM, Thorsteinsson SB, Minuth JN, Luchi RJ (1976). Vertebral osteomyelitis. Still a diagnostic pitfall. Arch. Intern. Med..

[11] Conan Y, Laurent E, Belin Y, Lacasse M, Amelot A, Mulleman D (2021). Large increase of vertebral osteomyelitis in France: A 2010–2019 cross-sectional study. Epidemiol. Infect..

[12] Thavarajasingam SG, Ponniah HS, Philipps R, Neuhoff J, Kramer A, Demetriades AK (2023). Increasing incidence of spondylodiscitis in England: An analysis of the national health service (NHS) hospital episode statistics from 2012 to 2021. Brain Spine.

[13] Lang S, Walter N, Schindler M, Baertl S, Szymski D, Loibl M (2023). The epidemiology of spondylodiscitis in Germany: A descriptive report of incidence rates, pathogens, in-hospital mortality, and hospital stays between 2010 and 2020. J. Clin. Med..

[14] Cheung WY, Luk KD (2012). Pyogenic spondylitis. Int. Orthop..

[15] Gouliouris T, Aliyu SH, Brown NM (2010). Spondylodiscitis: Update on diagnosis and management. J. Antimicrob. Chemother..

[16] Kehrer M, Pedersen C, Jensen TG, Lassen AT (2014). Increasing incidence of pyogenic spondylodiscitis: A 14-year population-based study. J. Infect..

[17] Santoro A, Bientinesi E, Monti D (2021). Immunosenescence and inflammaging in the aging process: Age-related diseases or longevity?. Ageing Res. Rev..

[18] Collard RM, Boter H, Schoevers RA, Oude Voshaar RC (2012). Prevalence of frailty in community-dwelling older persons: A systematic review. J. Am. Geriatr. Soc..

[19] Fried LP, Tangen CM, Walston J, Newman AB, Hirsch C, Gottdiener J (2001). Frailty in older adults: Evidence for a phenotype. J. Gerontol. A Biol. Sci. Med. Sci..

[20] IDF (2019). Diabetes Atlas.

[21] Harpaz R, Dahl RM, Dooling KL (2016). Prevalence of immunosuppression among US adults, 2013. JAMA.

[22] Heck VJ, Klug K, Prasse T, Oikonomidis S, Klug A, Himpe B (2023). Projections from surgical use models in Germany suggest a rising number of spinal fusions in patients 75 years and older will challenge healthcare systems worldwide. Clin. Orthop. Relat. Res..

[23] Kalseth J, Halvorsen T (2020). Health and care service utilisation and cost over the life-span: A descriptive analysis of population data. BMC Health Serv. Res..

[24] United Nations, D.o.E., P.D. Social Affairs, *World population ageing 2019: highlights (St/Esa/Ser. A/430)*. 2019, United Nations New York.

[25] Jackson KL, Rumley J, Griffith M, Agochukwu U, DeVine J (2020). Correlating psychological comorbidities and outcomes after spine surgery. Glob. Spine J..

[26] Aagaard T, Roed C, Dahl B, Obel N (2016). Long-term prognosis and causes of death after spondylodiscitis: A Danish nationwide cohort study. Infect. Dis. (Lond.).

[27] Bernard L, Dinh A, Ghout I, Simo D, Zeller V, Issartel B (2015). Antibiotic treatment for 6 weeks versus 12 weeks in patients with pyogenic vertebral osteomyelitis: An open-label, non-inferiority, randomised, controlled trial. Lancet.

[28] Deutsche Wirbelsäulengesellschaft e.V. (DWG), D.G.f.O.u.O.C.e.V.D., *S2k-Leitlinie Diagnostik und Therapie der Spondylodiszitis.* https://register.awmf.org/assets/guidelines/151-001l_S2k_Diagnostik-Therapie-Spondylodiszitis_2020-10.pdf, Version 1.0 (Accessed 26 August 2020).

[29] Bejon PA, Li HK, Rombach I, Walker S, Scarborough M (2019). The OVIVA trial. Lancet Infect. Dis..

[30] Li HK, Scarborough M, Zambellas R, Cooper C, Rombach I, Walker AS (2015). Oral versus intravenous antibiotic treatment for bone and joint infections (OVIVA): Study protocol for a randomised controlled trial. Trials.

