# Supplementary material for: Epidemiological trends of pyogenic spondylodiscitis in Germany: an EANS Spine Section Study
Source: Sci Rep. 2023 Nov 18;13:20225. doi: 10.1038/s41598-023-47341-z (PMC10657388; doi:10.1038/s41598-023-47341-z)
Supplement: Supplementary file 1 — Supplementary Tables. [file 41598_2023_47341_MOESM1_ESM.docx]

**Supplementary Material**

**Epidemiological trends of pyogenic spondylodiscitis in Germany:**

**An EANS Spine Section Study**

Andreas Kramer^1,8,* #^; Santhosh G. Thavarajasingam^2,3,4,8,#^; Jonathan Neuhoff^5,8^;

Hariharan Subbiah Ponniah^2,4^; Daniele S. C. Ramsay^2,4^; Andreas K. Demetriades^6,8^;

Benjamin M. Davies^3,8^; Ehab Shiban^7,8^; Florian Ringel^1,8^

^#^These authors have contributed equally to this work and share first authorship

**INSTITUTION:**

1. Department of Neurosurgery, University Medical Center Mainz, Mainz, Germany
2. Faculty of Medicine, Imperial College London, London,
3. United Kingdom Department of Academic Neurosurgery, Addenbroke‘s Hospital, Cambridge University Hospital NHS Healthcare Trust
4. Imperial Brain & Spine Initiative, Imperial College London, London,
5. Center for Spinal Surgery and Neurotraumatology, Berufsgenossenschaftliche Unfallklinik Frankfurt am Main, Germany
6. Edinburgh Spinal Surgery Outcome Studies Group, Department of Neurosurgery, Division of Clinical Neurosciences, NHS Lothian, Edinburgh University Hospitals, Edinburgh, United Kingdom
7. Department of Neurosurgery, Universitätsklinikum Augsburg, Augsburg, Germany
8. Spondylodiscitis Study Group, EANS Spine Section

^*^**Corresponding author:**

Andreas Kramer, MD

Department of Neurosurgery

University Medical Center Mainz

Johannes Gutenberg University

Langenbeckstraße. 1

55131 Mainz

Email: andreas.kramer@unimedizin-mainz.de

Phone: +49 6131 17 7331

ORCID: 0000-0003-0168-0178

**Supplementary Table S1:** Data extracted from the German Federal Statistical Office database for spondylodiscitis (ICD-10: M46.4 + M46.3+ M46.2) between 2005 and 2021.

| **Year** | **Age group** | **Diagnoses** | **Incidence** | **LOS** | **Regular DC** | **DC AMA** | **Death** | **Transfer TOH** | **DC to R/CH/H** | **Diagnoses w/ SF** | **Diagnoses w/ VBR** | **Diagnoses w/ CSR** |
| --- | --- | --- | --- | --- | --- | --- | --- | --- | --- | --- | --- | --- |
| 2005 | Age 0-19 | 64.0 | 0.077634 | 14.9 | 58 | 3 | 0 | 3 | 0 |  | 0 | 0 |
| 2005 | Age 20-29 | 44.0 | 0.053373 | 18.1 | 34 | 2 | 0 | 7 | 1 | 5 |  | 0 |
| 2005 | Age 30-39 | 199.0 | 0.241394 | 20.1 | 161 | 3 | 0 | 26 | 8 | 34 |  | 0 |
| 2005 | Age 40-49 | 366.0 | 0.443970 | 21.4 | 277 | 3 | 3 | 59 | 23 | 67 | 25 | 6 |
| 2005 | Age 50-59 | 621.0 | 0.753293 | 22.5 | 443 | 5 | 12 | 95 | 47 | 141 | 56 | 4 |
| 2005 | Age 60-69 | 1263.0 | 1.532061 | 27.3 | 810 | 9 | 38 | 248 | 143 | 253 | 119 | 8 |
| 2005 | Age 70-79 | 1336.0 | 1.620612 | 29.8 | 702 | 5 | 64 | 332 | 217 | 235 | 129 | 12 |
| 2005 | Age 80-89 | 545.0 | 0.661103 | 30.3 | 291 | 0 | 34 | 117 | 100 | 72 | 36 | 5 |
| 2005 | Age 90+ | 19.0 | 0.023048 | 35.4 | 9 | 0 | 2 | 1 | 7 |  | 0 | 0 |
| 2005 | Total | 4457 | 5.40648763 | 26.3 | 2785 | 30 | 153 | 888 | 546 | 811 | 375 | 35 |
| 2006 | Age 0-19 | 46 | 0.055883 | 17.7 | 44 | 2 | 0 | 0 | 0 | 3 |  |  |
| 2006 | Age 20-29 | 50 | 0.060742 | 32.2 | 39 | 2 | 0 | 5 | 3 | 10 |  | 0 |
| 2006 | Age 30-39 | 167 | 0.202879 | 18.8 | 133 | 6 | 0 | 22 | 6 | 34 | 4 | 0 |
| 2006 | Age 40-49 | 395 | 0.479864 | 22.2 | 315 | 8 | 3 | 43 | 25 | 81 | 28 |  |
| 2006 | Age 50-59 | 688 | 0.835815 | 29.2 | 480 | 4 | 9 | 130 | 59 | 159 | 53 | 5 |
| 2006 | Age 60-69 | 1411 | 1.714149 | 26.3 | 883 | 4 | 34 | 282 | 192 | 372 | 136 | 16 |
| 2006 | Age 70-79 | 1469 | 1.784610 | 30.5 | 816 | 5 | 80 | 327 | 232 | 314 | 112 | 11 |
| 2006 | Age 80-89 | 668 | 0.811518 | 28.7 | 347 | 2 | 37 | 154 | 126 | 99 | 32 | 5 |
| 2006 | Age 90+ | 32 | 0.038875 | 18.4 | 16 | 0 | 6 | 7 | 3 | 3 | 0 | 0 |
| 2006 | Total | 4926 | 5.984335 | 27.4 | 3073 | 33 | 169 | 970 | 646 | 1075 | 370 | 40 |
| 2007 | Age 0-19 | 54.0 | 0.065679 | 18.4 | 49 | 1 | 0 | 4 | 0 | 3 |  | 0 |
| 2007 | Age 20-29 | 51.0 | 0.062030 | 16.6 | 44 | 0 | 0 | 7 | 0 | 8 |  |  |
| 2007 | Age 30-39 | 168.0 | 0.204335 | 21.3 | 135 | 8 | 1 | 19 | 4 | 37 | 12 | 0 |
| 2007 | Age 40-49 | 417.0 | 0.507189 | 18.5 | 322 | 7 | 2 | 62 | 21 | 114 | 26 | 5 |
| 2007 | Age 50-59 | 733.0 | 0.891534 | 25.1 | 532 | 7 | 8 | 128 | 53 | 194 | 55 |  |
| 2007 | Age 60-69 | 1488.0 | 1.809826 | 27.3 | 922 | 7 | 48 | 328 | 173 | 388 | 135 | 18 |
| 2007 | Age 70-79 | 1794.0 | 2.182008 | 30.2 | 998 | 16 | 84 | 428 | 256 | 403 | 140 | 18 |
| 2007 | Age 80-89 | 797.0 | 0.969376 | 28.9 | 385 | 4 | 74 | 164 | 165 | 121 | 42 |  |
| 2007 | Age 90+ | 38.0 | 0.046219 | 21.7 | 18 | 0 | 4 | 8 | 8 | 3 | 0 | 0 |
| 2007 | Total | 5540.0 | 6.738197 | 26.8 | 3405 | 50 | 221 | 1148 | 680 | 1271 | 415 | 46 |
| 2008 | Age 0-19 | 56.0 | 0.068112 | 13.6 | 54 | 1 | 0 | 0 | 1 |  |  |  |
| 2008 | Age 20-29 | 47.0 | 0.057315 | 15.0 | 36 | 1 | 0 | 6 | 4 | 14 |  | 0 |
| 2008 | Age 30-39 | 138.0 | 0.168288 | 22.1 | 116 | 5 | 0 | 12 | 3 | 33 | 10 | 0 |
| 2008 | Age 40-49 | 481.0 | 0.586569 | 20.0 | 355 | 8 | 4 | 81 | 24 | 132 | 29 |  |
| 2008 | Age 50-59 | 806.0 | 0.982899 | 25.0 | 582 | 11 | 12 | 127 | 71 | 194 | 61 |  |
| 2008 | Age 60-69 | 1548.0 | 1.887751 | 30.6 | 974 | 7 | 43 | 332 | 183 | 462 | 135 | 13 |
| 2008 | Age 70-79 | 2029.0 | 2.474319 | 28.5 | 1068 | 4 | 98 | 524 | 327 | 513 | 170 | 15 |
| 2008 | Age 80-89 | 882.0 | 1.075579 | 28.0 | 432 | 3 | 80 | 207 | 156 | 159 | 43 | 9 |
| 2008 | Age 90+ | 45.0 | 0.054876 | 31.9 | 21 | 1 | 10 | 5 | 8 |  | 0 | 0 |
| 2008 | Total | 6032.0 | 7.355886 | 27.0 | 3638 | 41 | 247 | 1294 | 777 | 1513 | 452 | 43 |
| 2009 | Age 0-19 | 52.0 | 0.063568 | 13.8 | 50 | 1 | 0 | 1 | 0 | 4 | 0 | 0 |
| 2009 | Age 20-29 | 44.0 | 0.053788 | 17.3 | 35 | 0 | 0 | 8 | 0 | 5 |  | 0 |
| 2009 | Age 30-39 | 147.0 | 0.179702 | 18.4 | 120 | 3 | 1 | 16 | 7 | 22 | 7 | 0 |
| 2009 | Age 40-49 | 435.0 | 0.531770 | 19.3 | 355 | 6 | 1 | 58 | 13 | 105 | 22 |  |
| 2009 | Age 50-59 | 826.0 | 1.009752 | 24.6 | 584 | 9 | 19 | 152 | 58 | 200 | 68 | 9 |
| 2009 | Age 60-69 | 1459.0 | 1.783569 | 27.0 | 933 | 12 | 33 | 309 | 164 | 289 | 115 | 10 |
| 2009 | Age 70-79 | 2193.0 | 2.680855 | 31.1 | 1217 | 12 | 107 | 517 | 322 | 314 | 164 | 13 |
| 2009 | Age 80-89 | 1046.0 | 1.278693 | 29.5 | 497 | 12 | 81 | 248 | 203 | 103 | 48 |  |
| 2009 | Age 90+ | 40.0 | 0.048898 | 29.4 | 15 | 0 | 7 | 7 | 10 | 0 |  | 0 |
| 2009 | Total | 6242.0 | 7.630596 | 27.0 | 3806 | 55 | 249 | 1316 | 777 | 1042 | 426 | 41 |
| 2010 | Age 0-19 | 51.0 | 0.062384 | 16.0 | 42 | 1 | 0 | 7 | 1 | 4 |  | 0 |
| 2010 | Age 20-29 | 48.0 | 0.058714 | 10.6 | 45 | 1 | 0 | 2 | 0 | 10 |  | 0 |
| 2010 | Age 30-39 | 138.0 | 0.168804 | 18.0 | 113 | 2 | 0 | 18 | 3 | 43 | 8 |  |
| 2010 | Age 40-49 | 433.0 | 0.529653 | 22.6 | 333 | 6 | 3 | 59 | 28 | 120 | 24 |  |
| 2010 | Age 50-59 | 940.0 | 1.149825 | 22.4 | 690 | 10 | 16 | 142 | 80 | 279 | 63 | 7 |
| 2010 | Age 60-69 | 1583.0 | 1.936353 | 28.8 | 1025 | 11 | 61 | 293 | 191 | 494 | 119 | 15 |
| 2010 | Age 70-79 | 2467.0 | 3.017678 | 31.0 | 1330 | 17 | 124 | 589 | 395 | 686 | 195 | 25 |
| 2010 | Age 80-89 | 1215.0 | 1.486209 | 26.7 | 588 | 5 | 104 | 282 | 228 | 239 | 61 | 6 |
| 2010 | Age 90+ | 62.0 | 0.075839 | 23.0 | 33 | 0 | 6 | 12 | 11 | 6 |  | 0 |
| 2010 | Total | 6937.0 | 8.485461 | 27.2 | 4199 | 53 | 314 | 1404 | 937 | 1881 | 474 | 55 |
| 2011 | Age 0-19 | 66.0 | 0.082163 | 13.1 | 59 | 2 | 0 | 5 | 0 |  |  | 0 |
| 2011 | Age 20-29 | 40.0 | 0.049796 | 12.5 | 31 | 0 | 0 | 9 | 0 | 9 |  | 0 |
| 2011 | Age 30-39 | 144.0 | 0.179265 | 19.7 | 118 | 5 | 1 | 13 | 5 | 46 | 13 |  |
| 2011 | Age 40-49 | 411.0 | 0.511653 | 18.0 | 321 | 8 | 3 | 59 | 19 | 134 | 32 |  |
| 2011 | Age 50-59 | 919.0 | 1.144061 | 23.5 | 653 | 10 | 13 | 158 | 82 | 281 | 78 | 8 |
| 2011 | Age 60-69 | 1584.0 | 1.971918 | 27.0 | 1024 | 11 | 42 | 332 | 165 | 525 | 126 | 22 |
| 2011 | Age 70-79 | 2606.0 | 3.244203 | 28.6 | 1413 | 25 | 117 | 622 | 421 | 720 | 184 | 27 |
| 2011 | Age 80-89 | 1279.0 | 1.592224 | 27.0 | 585 | 8 | 119 | 324 | 233 | 283 | 53 | 12 |
| 2011 | Age 90+ | 84.0 | 0.104571 | 30.7 | 49 | 1 | 12 | 13 | 9 |  | 4 | 0 |
| 2011 | Total | 7133.0 | 8.879854 | 25.6 | 4253 | 70 | 307 | 1535 | 934 | 2009 | 493 | 75 |
| 2012 | Age 0-19 | 58.0 | 0.072028 | 18.0 | 54 | 3 | 0 | 1 | 0 |  | 0 | 0 |
| 2012 | Age 20-29 | 48.0 | 0.059610 | 14.0 | 40 | 2 | 0 | 5 | 1 | 13 |  |  |
| 2012 | Age 30-39 | 164.0 | 0.203667 | 14.7 | 130 | 3 | 0 | 24 | 6 | 44 | 16 | 0 |
| 2012 | Age 40-49 | 488.0 | 0.606032 | 23.7 | 374 | 8 | 2 | 75 | 28 | 170 | 38 |  |
| 2012 | Age 50-59 | 968.0 | 1.202130 | 24.5 | 711 | 13 | 13 | 150 | 77 | 301 | 69 | 11 |
| 2012 | Age 60-69 | 1576.0 | 1.957187 | 31.4 | 1025 | 15 | 52 | 280 | 199 | 524 | 142 | 14 |
| 2012 | Age 70-79 | 2882.0 | 3.579068 | 28.5 | 1612 | 9 | 119 | 678 | 454 | 824 | 234 | 34 |
| 2012 | Age 80-89 | 1367.0 | 1.697636 | 31.8 | 667 | 2 | 112 | 309 | 272 | 267 | 51 | 8 |
| 2012 | Age 90+ | 90.0 | 0.111768 | 14.5 | 39 | 1 | 16 | 10 | 24 |  |  | 0 |
| 2012 | Total | 7641.0 | 9.489126 | 28.0 | 4652 | 56 | 314 | 1532 | 1061 | 2155 | 553 | 69 |
| 2013 | Age 0-19 | 66.0 | 0.081716 | 14.5 | 61 | 1 | 0 | 3 | 1 |  | 3 |  |
| 2013 | Age 20-29 | 56.0 | 0.069335 | 15.4 | 42 | 4 | 0 | 9 | 1 |  | 4 |  |
| 2013 | Age 30-39 | 161.0 | 0.199338 | 17.2 | 130 | 7 | 0 | 19 | 4 | 48 |  | 0 |
| 2013 | Age 40-49 | 456.0 | 0.564584 | 18.8 | 346 | 6 | 3 | 71 | 29 | 151 | 52 | 9 |
| 2013 | Age 50-59 | 1012.0 | 1.252980 | 22.6 | 744 | 14 | 17 | 172 | 64 | 288 | 98 | 11 |
| 2013 | Age 60-69 | 1584.0 | 1.961186 | 26.9 | 1014 | 13 | 40 | 305 | 205 | 499 | 154 | 17 |
| 2013 | Age 70-79 | 2995.0 | 3.708176 | 27.6 | 1642 | 18 | 161 | 741 | 424 | 795 | 216 | 36 |
| 2013 | Age 80-89 | 1558.0 | 1.928995 | 25.6 | 713 | 7 | 151 | 380 | 298 | 339 | 74 | 9 |
| 2013 | Age 90+ | 124.0 | 0.153527 | 27.5 | 56 | 1 | 21 | 23 | 23 | 12 |  | 0 |
| 2013 | Total | 8012.0 | 9.919836 | 25.0 | 4748 | 71 | 393 | 1723 | 1049 | 2143 | 609 | 84 |
| 2014 | Age 0-19 | 58 | 0.071431 | 17.1 | 54 | 1 | 0 | 3 | 0 | 20 |  |  |
| 2014 | Age 20-29 | 51 | 0.062810 | 13.6 | 40 | 1 | 0 | 7 | 3 | 15 |  | 0 |
| 2014 | Age 30-39 | 183 | 0.225376 | 20.4 | 147 | 12 | 0 | 19 | 4 | 34 | 7 |  |
| 2014 | Age 40-49 | 429 | 0.528341 | 15.5 | 335 | 12 | 2 | 57 | 23 | 97 | 25 |  |
| 2014 | Age 50-59 | 1004 | 1.236491 | 23.5 | 706 | 16 | 13 | 188 | 80 | 250 | 94 | 23 |
| 2014 | Age 60-69 | 1649 | 2.030850 | 28.4 | 1070 | 42 | 57 | 326 | 150 | 280 | 154 | 23 |
| 2014 | Age 70-79 | 3372 | 4.152835 | 26.6 | 1851 | 19 | 182 | 821 | 486 | 467 | 246 | 26 |
| 2014 | Age 80-89 | 1697 | 2.089965 | 27.6 | 862 | 4 | 137 | 376 | 307 | 187 | 76 | 15 |
| 2014 | Age 90+ | 134 | 0.165030 | 28.8 | 68 | 0 | 18 | 21 | 26 | 6 | 4 | 0 |
| 2014 | Total | 8577 | 10.563128 | 25.6 | 5133 | 107 | 409 | 1818 | 1079 | 1356 | 611 | 91 |
| 2015 | Age 0-19 | 84.0 | 0.102220 | 23.3 | 78 | 3 | 0 | 3 | 0 | 4 |  | 0 |
| 2015 | Age 20-29 | 56.0 | 0.068147 | 16.2 | 42 | 4 | 0 | 10 | 0 | 10 |  | 0 |
| 2015 | Age 30-39 | 189.0 | 0.229995 | 14.1 | 143 | 6 | 1 | 34 | 4 | 52 | 13 |  |
| 2015 | Age 40-49 | 440.0 | 0.535438 | 16.2 | 346 | 13 | 5 | 64 | 10 | 135 | 43 | 7 |
| 2015 | Age 50-59 | 1132.0 | 1.377536 | 17.4 | 817 | 17 | 27 | 200 | 69 | 345 | 106 | 7 |
| 2015 | Age 60-69 | 1669.0 | 2.031014 | 22.0 | 1095 | 22 | 52 | 331 | 167 | 508 | 136 | 11 |
| 2015 | Age 70-79 | 3061.0 | 3.724946 | 24.5 | 1645 | 17 | 186 | 754 | 444 | 802 | 259 | 29 |
| 2015 | Age 80-89 | 1912.0 | 2.326722 | 25.5 | 921 | 3 | 186 | 489 | 302 | 399 | 107 | 19 |
| 2015 | Age 90+ | 159.0 | 0.193488 | 23.3 | 74 | 2 | 21 | 31 | 30 | 17 | 4 |  |
| 2015 | Total | 8702.0 | 10.589507 | 17.3 | 5161 | 87 | 478 | 1916 | 1026 | 2272 | 673 | 77 |
| 2016 | Age 0-19 | 88.0 | 0.106639 | 24.4 | 81 | 0 | 0 | 6 | 1 | 8 |  |  |
| 2016 | Age 20-29 | 56.0 | 0.067861 | 12.3 | 42 | 2 | 0 | 10 | 2 | 19 | 6 | 0 |
| 2016 | Age 30-39 | 171.0 | 0.207218 | 12.3 | 138 | 8 | 1 | 18 | 5 | 52 | 12 | 0 |
| 2016 | Age 40-49 | 404.0 | 0.489568 | 18.3 | 311 | 7 | 1 | 67 | 15 | 104 | 26 |  |
| 2016 | Age 50-59 | 1152.0 | 1.395997 | 16.1 | 830 | 22 | 22 | 193 | 83 | 258 | 95 | 8 |
| 2016 | Age 60-69 | 1891.0 | 2.291520 | 21.5 | 1236 | 22 | 59 | 404 | 164 | 311 | 148 | 12 |
| 2016 | Age 70-79 | 3244.0 | 3.931089 | 23.6 | 1791 | 20 | 190 | 818 | 409 | 455 | 239 | 34 |
| 2016 | Age 80-89 | 2145.0 | 2.599318 | 28.0 | 1037 | 12 | 207 | 511 | 371 | 193 | 99 | 20 |
| 2016 | Age 90+ | 180.0 | 0.218125 | 26.4 | 75 | 3 | 33 | 40 | 29 | 9 |  | 0 |
| 2016 | Total | 9331.0 | 11.307335 | 23.3 | 5541 | 96 | 513 | 2067 | 1079 | 1409 | 628 | 78 |
| 2017 | Age 0-19 | 93.0 | 0.112329 | 23.2 | 83 | 4 | 0 | 5 | 1 | 22 |  |  |
| 2017 | Age 20-29 | 64.0 | 0.077302 | 13.4 | 50 | 4 | 0 | 7 | 3 | 8 | 5 | 0 |
| 2017 | Age 30-39 | 162.0 | 0.195670 | 18.6 | 123 | 8 | 0 | 26 | 4 | 43 | 8 | 0 |
| 2017 | Age 40-49 | 451.0 | 0.544736 | 14.5 | 343 | 11 | 5 | 73 | 19 | 119 | 34 | 0 |
| 2017 | Age 50-59 | 1169.0 | 1.411966 | 19.1 | 865 | 24 | 19 | 200 | 57 | 250 | 77 |  |
| 2017 | Age 60-69 | 1891.0 | 2.284027 | 20.0 | 1214 | 11 | 65 | 393 | 205 | 316 | 137 | 17 |
| 2017 | Age 70-79 | 3375.0 | 4.076464 | 25.2 | 1812 | 23 | 216 | 887 | 422 | 371 | 196 | 21 |
| 2017 | Age 80-89 | 2460.0 | 2.971289 | 25.0 | 1166 | 14 | 257 | 606 | 409 | 182 | 103 | 8 |
| 2017 | Age 90+ | 179.0 | 0.216204 | 24.0 | 75 | 0 | 34 | 31 | 39 | 12 |  | 0 |
| 2017 | Total | 9844.0 | 11.889987 | 18.3 | 5731 | 99 | 596 | 2228 | 1159 | 1323 | 565 | 54 |
| 2018 | Age 0-19 | 89.0 | 0.107204 | 13.7 | 80 | 4 | 0 | 5 | 0 | 9 | 0 |  |
| 2018 | Age 20-29 | 68.0 | 0.081909 | 16.7 | 56 | 3 | 0 | 7 | 2 | 18 | 3 | 0 |
| 2018 | Age 30-39 | 178.0 | 0.214408 | 13.7 | 139 | 3 | 0 | 30 | 4 | 51 | 8 | 3 |
| 2018 | Age 40-49 | 468.0 | 0.563725 | 16.1 | 363 | 12 | 7 | 71 | 11 | 91 | 46 | 3 |
| 2018 | Age 50-59 | 1185.0 | 1.427380 | 19.8 | 852 | 20 | 24 | 218 | 69 | 302 | 74 |  |
| 2018 | Age 60-69 | 1964.0 | 2.365717 | 25.3 | 1291 | 24 | 68 | 412 | 163 | 323 | 146 | 15 |
| 2018 | Age 70-79 | 3181.0 | 3.831643 | 25.6 | 1751 | 25 | 191 | 799 | 401 | 365 | 225 | 20 |
| 2018 | Age 80-89 | 2426.0 | 2.922215 | 23.3 | 1140 | 10 | 251 | 607 | 403 | 239 | 117 | 15 |
| 2018 | Age 90+ | 207.0 | 0.249340 | 19.0 | 93 | 0 | 36 | 38 | 38 | 15 | 3 | 0 |
| 2018 | Total | 9766.0 | 11.763542 | 22.8 | 5765 | 101 | 577 | 2187 | 1091 | 1413 | 622 | 61 |
| 2019 | Age 0-19 | 86 | 0.103407 | 16.2 | 82 | 0 | 0 | 4 | 0 | 8 |  | 0 |
| 2019 | Age 20-29 | 65 | 0.078156 | 13.6 | 43 | 6 | 1 | 13 | 1 | 11 |  | 0 |
| 2019 | Age 30-39 | 186 | 0.223647 | 13.0 | 154 | 9 | 0 | 17 | 2 | 41 | 15 | 3 |
| 2019 | Age 40-49 | 438 | 0.526653 | 16.5 | 339 | 8 | 3 | 71 | 11 | 99 | 37 | 4 |
| 2019 | Age 50-59 | 1176 | 1.414027 | 21.9 | 849 | 21 | 16 | 231 | 56 | 263 | 104 | 9 |
| 2019 | Age 60-69 | 2018 | 2.426452 | 22.1 | 1315 | 20 | 73 | 442 | 160 | 334 | 162 | 13 |
| 2019 | Age 70-79 | 3266 | 3.927052 | 27.0 | 1839 | 26 | 182 | 823 | 379 | 382 | 202 | 18 |
| 2019 | Age 80-89 | 2658 | 3.195990 | 22.7 | 1262 | 16 | 275 | 691 | 398 | 265 | 110 | 15 |
| 2019 | Age 90+ | 228 | 0.274148 | 27.4 | 114 | 2 | 35 | 40 | 37 | 14 |  | 0 |
| 2019 | Total | 10121 | 12.169533 | 22.4 | 5997 | 108 | 585 | 2332 | 1044 | 1417 | 634 | 62 |
| 2020 | Age 0-19 | 55 | 0.066142 | 23.0 | 50 | 0 | 0 | 5 | 0 | 9 | 0 |  |
| 2020 | Age 20-29 | 57 | 0.068547 | 8.1 | 49 | 2 | 0 | 6 | 0 | 15 | 6 |  |
| 2020 | Age 30-39 | 206 | 0.247730 | 17.1 | 165 | 7 | 1 | 25 | 5 | 48 | 15 |  |
| 2020 | Age 40-49 | 413 | 0.496663 | 13.1 | 314 | 17 | 3 | 62 | 13 | 93 | 28 | 5 |
| 2020 | Age 50-59 | 1090 | 1.310805 | 19.2 | 804 | 18 | 24 | 171 | 66 | 282 | 99 | 5 |
| 2020 | Age 60-69 | 1857 | 2.233178 | 18.7 | 1209 | 26 | 74 | 382 | 156 | 341 | 156 | 17 |
| 2020 | Age 70-79 | 2908 | 3.497082 | 24.1 | 1670 | 26 | 191 | 686 | 321 | 358 | 185 | 17 |
| 2020 | Age 80-89 | 2716 | 3.266188 | 24.6 | 1322 | 18 | 295 | 666 | 399 | 235 | 115 | 12 |
| 2020 | Age 90+ | 252 | 0.303048 | 24.8 | 103 | 0 | 43 | 52 | 52 | 16 | 5 | 0 |
| 2020 | Total | 9554 | 11.489383 | 21.3 | 5686 | 114 | 631 | 2055 | 1012 | 1397 | 609 | 61 |
| 2021 | Age 0-19 | 44 | 0.052861 | 15.6 | 39 | 1 | 0 | 3 | 1 | 3 |  |  |
| 2021 | Age 20-29 | 44 | 0.052861 | 10.2 | 33 | 3 | 0 | 7 | 0 | 5 |  | 0 |
| 2021 | Age 30-39 | 153 | 0.183812 | 15.8 | 113 | 6 | 0 | 30 | 2 | 41 | 9 | 0 |
| 2021 | Age 40-49 | 388 | 0.466138 | 15.1 | 303 | 9 | 6 | 56 | 9 | 115 | 29 | 0 |
| 2021 | Age 50-59 | 1055 | 1.267463 | 20.6 | 747 | 17 | 29 | 198 | 57 | 302 | 90 | 0 |
| 2021 | Age 60-69 | 1909 | 2.293448 | 24.7 | 1219 | 35 | 83 | 402 | 166 | 510 | 122 | 0 |
| 2021 | Age 70-79 | 2686 | 3.226926 | 24.3 | 1499 | 27 | 199 | 635 | 317 | 603 | 131 | 0 |
| 2021 | Age 80-89 | 2618 | 3.145231 | 22.4 | 1237 | 20 | 316 | 653 | 381 | 456 | 100 | 0 |
| 2021 | Age 90+ | 270 | 0.324374 | 22.5 | 118 | 0 | 51 | 40 | 60 | 20 | 5 | 0 |
| 2021 | Total | 9167 | 11.013115 | 22.4 | 5308 | 118 | 684 | 2024 | 993 | 2055 | 490 | 0 |

A table showing the data extracted from the German Federal Statistical Office database for spondylodiscitis (ICD-10: M46.4 + M46.3+ M46.2)  between 2005 and 20201. The following variables were extracted: Year, Age, number of diagnoses for spondylodiscitis (‘Diagnoses’), Population, Incidence, length of stay (‘LOS’), regular discharge (‘DC’), DC against medical advise, number of deaths after spondylodiscitis hospital diagnosis (‘Death’), transfer to other hospital, DC to rehab/care home/hospice, the proportion of discharges being deaths due to patients dying (‘Proportion of DC being Death’), diagnoses after spinal fusion (‘Diagnoses w/ SF), diagnoses after vertebral body replacement (‘Diagnoses w/ VBR’), diagnoses after complex spinal reconstruction (‘Diagnoses w/ CSR’). DC, discharge.

**Supplementary Table S2:** Multivariate linear regression analysis of the relationship between the number of diagnoses of spondylodiscitis (ICD-10: M46.4 + M46.3+ M46.2, 2005 to 2021) and various factors.

| **Linear regression** | | | | |
| --- | --- | --- | --- | --- |
| **Call: lm(formula=lm(formula=Diagnoses ~ as.factor(Year) + as.factor(Age) + Incidence + LOS + `Regular DC` + `DC against medical advise` + Death + `Transfer to other hospital` + `DC to rehab/care home/hospice` + `Proportion of DC being Death` + `Death:Diagnoses` + `Diagnoses w/spinal fusion` + `Diagnoses w/vertebral body replacement` + `Diagnoses w/complex spinal reconstruction`, data=GermanSpondy)** | | | | |
|  | Estimate. | Std. Error. | t value | Pr(>\|t\|) |
| **(Intercept)** | 3.185555 | 4.506041 | 0.707 | 0.48194 |
| **as.factor(Year)2006** | -2.241527 | 1.990369 | -1.126 | 0.26393 |
| **as.factor(Year)2007** | -1.806987 | 2.061459 | -0.877 | 0.38373 |
| **as.factor(Year)2008** | -3.994366 | 2.213559 | -1.804 | 0.07545 . |
| **as.factor(Year)2009** | -2.395150 | 2.141321 | -1.119 | 0.26716 |
| **as.factor(Year)2010** | 0.135259 | 2.506160 | 0.054 | 0.95711 |
| **as.factor(Year)2011** | 1.220951 | 2.522396 | 0.484 | 0.62987 |
| **as.factor(Year)2012** | -1.302755 | 2.365881 | -0.551 | 0.58363 |
| **as.factor(Year)2013** | -4.072571 | 2.204083 | -1.848 | 0.06887 . |
| **as.factor(Year)2014** | -2.361408 | 2.352864 | -1.004 | 0.31901 |
| **as.factor(Year)2015** | -2.621054 | 2.488575 | -1.053 | 0.29586 |
| **as.factor(Year)2016** | -3.192588 | 2.357851 | -1.354 | 0.18008 |
| **as.factor(Year)2017** | -5.117651 | 2.423842 | -2.111 | 0.03831 * |
| **as.factor(Year)2018** | -1.564501 | 2.229181 | -0.702 | 0.48511 |
| **as.factor(Year)2019** | 1.143636 | 2.306910 | 0.496 | 0.62163 |
| **as.factor(Year)2020** | 0.924488 | 2.307777 | 0.401 | 0.68994 |
| **as.factor(Year)2021** | -1.707818 | 2.408050 | -0.709 | 0.48055 |
| **as.factor(Age)Age 20-29** | -1.154185 | 4.253284 | 0.271 | 0.78691 |
| **as.factor(Age)Age 30-39** | 2.910943 | 3.832563 | 0.760 | 0.45009 |
| **as.factor(Age)Age 40-49** | 5.902271 | 4.081195 | 1.446 | 0.15258 |
| **as.factor(Age)Age 50-59** | 10.050817 | 4.776193 | 2.104 | 0.03894 * |
| **as.factor(Age)Age 60-69** | 14.644553 | 5.631303 | 2.601 | 0.01135 * |
| **as.factor(Age)Age 70-79** | 16.120566 | 6.343115 | 2.541 | 0.01326 * |
| **as.factor(Age)Age 80-89** | 7.652141 | 6.108095 | 1.253 | 0.21445 |
| **as.factor(Age)Age 90+** | 3.260681 | 8.480028 | 0.385 | 0.70176 |
| **as.factor(Age)Total** | 62.436768 | 10.050305 | 6.212 | 3.29e-08 *** |
| **Incidence** | 58.783429 | 24.421743 | 2.407 | 0.01872 * |
| **LOS** | 0.008406 | 0.173385 | 0.048 | 0.96147 |
| **`Regular DC`** | 0.916220 | 0.029535 | 31.021 | < 2e-16 *** |
| **`DC against medical advise`** | -0.864386 | 0.023104 | 37.412 | < 2e-16 *** |
| **Death** | 1.043180 | 0.029186 | 35.743 | < 2e-16 *** |
| **`Transfer to other hospital`** | 0.946962 | 0.031674 | 29.897 | < 2e-16 *** |
| **`DC to rehab/care home/hospice`** | 0.875634 | 0.043517 | 20.122 | < 2e-16 *** |
| **`Proportion of DC being Death`** | 10.586414 | 15.954410 | 0.664 | 0.50916 |
| **`Death:Diagnoses`** | -42.347810 | 49.079277 | -0.863 | 0.39117 |
| **`Diagnoses w/spinal fusion`** | -0.016047 | 0.003482 | -4.609 | 1.77e-05 *** |
| **`Diagnoses w/vertebral body replacement`** | 0.103274 | 0.030130 | 3.428 | 0.00102 ** |
| **`Diagnoses w/complex spinal reconstruction`** | -0.145031 | 0.089918 | -1.613 | 0.11126 |
| **---** | | | | |
| **Signif. codes:  0 ‘***’ 0.001 ‘**’ 0.01 ‘*’ 0.05 ‘.’ 0.1 ‘ ’ 1** | | | | |
|  | | | | |
| **Residual standard error: 3.233 on 70 degrees of freedom** | | | | |
| **(62 observations deleted due to missingness)** | | | | |
| **Multiple R-squared:  1,** **Adjusted R-squared:  1** | | | | |
| **F-statistic:  1.909e+06 on 37 and 70 DF,  p-value:<2.2e-16** | | | | |

This table presents the results of a linear regression analysis of the relationship between the number of diagnoses of spondylodiscitis (ICD-10: M46.4 + M46.3+ M46.2, 2005 to 2021) and various factors including year, age, incidence, length of stay (LOS), types of discharge, death, transfer to other hospital, proportion of discharge being death, diagnoses with spinal fusion, vertebral body replacement, and complex spinal reconstruction. The table provides the coefficient estimates, standard errors, t-values, and p-values for each factor. The results are presented for each year from 2006 to 2021 (2005 is the intercept) and for each age group ranging from 20-29 to 90 and above, as well as a total row for all age groups combined. The table highlights statistically significant values using asterisks, where *** denotes a p-value less than 0.001, ** denotes a p-value less than 0.01, and * denotes a p-value less than 0.05. The table also provides information on the residual standard error, multiple R-squared, adjusted R-squared, and F-statistic for the overall regression model. The analysis involved 37 degrees of freedom and excluded 62 observations due to missing data.

**Supplementary Table S3:** Univariate linear regression analysis of the relationship between the number of diagnoses of spondylodiscitis (ICD-10: M46.4 + M46.3+ M46.2, 2005 to 2021) and various factors.

| **"Age group Age 0-19"** |
| --- |
|  |
| **Call:** |
| **lm(formula=Diagnoses ~ Year, data=df)** |
|  |
| Residuals: |
| Min      1Q  Median      3Q     Max |
| -32.490  -8.694  -2.297  12.309  22.108 |
|  |
| **Coefficients:** |
| **Estimate Std. Error t value Pr(>\|t\|)** |
| **(Intercept) -2751.9191  1513.6766  -1.818   0.0891 .** |
| **Year            1.3995     0.7519   1.861   0.0824 .** |
| **---** |
| **Signif. codes:  0 ‘***’ 0.001 ‘**’ 0.01 ‘*’ 0.05 ‘.’ 0.1 ‘ ’ 1** |
|  |
| Residual standard error: 15.19 on 15 degrees of freedom |
| Multiple R-squared:  0.1876, |
| F-statistic: 3.464 on 1 and 15 DF,  p-value: 0.08243 |
|  |
| **"Age group Age 20-29"** |
|  |
| **Call:** |
| **lm(formula=Diagnoses ~ Year, data=df)** |
|  |
| Residuals: |
| Min      1Q  Median      3Q     Max |
| -15.686  -2.218  -0.674   4.174  11.086 |
|  |
| **Coefficients:** |
| **Estimate Std. Error t value Pr(>\|t\|)** |
| **(Intercept) -1807.7574   676.4926  -2.672   0.0174 *** |
| **Year            0.9240     0.3361   2.750   0.0149 *** |
| **---** |
| **Signif. codes:  0 ‘***’ 0.001 ‘**’ 0.01 ‘*’ 0.05 ‘.’ 0.1 ‘ ’ 1** |
|  |
| Residual standard error: 6.788 on 15 degrees of freedom |
| Multiple R-squared:  0.3351, |
| F-statistic:  7.56 on 1 and 15 DF,  p-value: 0.0149 |
|  |
| **"Age group Age 30-39"** |
|  |
| **Call:** |
| **lm(formula=Diagnoses ~ Year, data=df)** |
|  |
| Residuals: |
| Min      1Q  Median      3Q     Max |
| -26.346 -16.167  -0.419  11.044  40.549 |
|  |
| **Coefficients:** |
| **Estimate Std. Error t value Pr(>\|t\|)** |
| **(Intercept) -2205.2868  2006.5328  -1.099    0.289** |
| **Year            1.1789     0.9968   1.183    0.255** |
| **---** |
| **Signif. codes:  0 ‘***’ 0.001 ‘**’ 0.01 ‘*’ 0.05 ‘.’ 0.1 ‘ ’ 1** |
| Multiple R-squared:  0.0853, |
| F-statistic: 1.399 on 1 and 15 DF,  p-value: 0.2553 |
|  |
| **"Age group Age 40-49"** |
|  |
| **Call:** |
| **lm(formula=Diagnoses ~ Year, data=df)** |
|  |
| Residuals: |
| Min      1Q  Median      3Q     Max |
| -57.529 -22.993   2.838  17.500  58.654 |
|  |
| **Coefficients:** |
| **Estimate Std. Error t value Pr(>\|t\|)** |
| **(Intercept) -1242.3897  3357.2592  -0.370    0.717** |
| **Year            0.8309     1.6678   0.498    0.626** |
|  |
| **Residual standard error: 33.69 on 15 degrees of freedom** |
| **Multiple R-squared:  0.01628,** |
| **F-statistic: 0.2482 on 1 and 15 DF,  p-value: 0.6256** |
|  |
| **"Age group Age 50-59"** |
|  |
| **Call:** |
| **lm(formula=Diagnoses ~ Year, data=df)** |
|  |
| Residuals: |
| Min      1Q  Median      3Q     Max |
| -175.14  -40.46   11.10   52.72   97.58 |
|  |
| **Coefficients:** |
| **Estimate Std. Error t value Pr(>\|t\|)** |
| **(Intercept) -64695.081   7574.651  -8.541 3.81e-07 ***** |
| **Year            32.620      3.763   8.669 3.16e-07 ***** |
| **---** |
| **Signif. codes:  0 ‘***’ 0.001 ‘**’ 0.01 ‘*’ 0.05 ‘.’ 0.1 ‘ ’ 1** |
|  |
| Residual standard error: 76.01 on 15 degrees of freedom |
| Multiple R-squared:  0.8336, |
| F-statistic: 75.15 on 1 and 15 DF,  p-value: 3.159e-07 |
|  |
| **"Age group Age 60-69"** |
|  |
| **Call:** |
| **lm(formula=Diagnoses ~ Year, data=df)** |
|  |
| Residuals: |
| Min      1Q  Median      3Q     Max |
| -92.439 -78.907  -2.681  62.544 108.868 |
|  |
| **Coefficients:** |
| **Estimate Std. Error t value Pr(>\|t\|)** |
| **(Intercept) -79469.434   7772.831  -10.22 3.73e-08 ***** |
| **Year            40.306      3.861   10.44 2.83e-08 ***** |
| **---** |
| **Signif. codes:  0 ‘***’ 0.001 ‘**’ 0.01 ‘*’ 0.05 ‘.’ 0.1 ‘ ’ 1** |
|  |
| Residual standard error: 77.99 on 15 degrees of freedom |
| Multiple R-squared:  0.879, |
| F-statistic:   109 on 1 and 15 DF,  p-value: 2.833e-08 |
|  |
| **"Age group Age 70-79"** |
|  |
| **Call:** |
| **lm(formula=Diagnoses ~ Year, data=df)** |
|  |
| Residuals: |
| Min      1Q  Median      3Q     Max |
| -814.31 -199.12    3.66  281.97  625.28 |
|  |
| **Coefficients:** |
| **Estimate Std. Error t value Pr(>\|t\|)** |
| **(Intercept) -214074.21   38374.36  -5.579 5.27e-05 ***** |
| **Year            107.66      19.06   5.647 4.64e-05 ***** |
| **---** |
| **Signif. codes:  0 ‘***’ 0.001 ‘**’ 0.01 ‘*’ 0.05 ‘.’ 0.1 ‘ ’ 1** |
|  |
| Residual standard error: 385.1 on 15 degrees of freedom |
| Multiple R-squared:  0.6801, |
| F-statistic: 31.89 on 1 and 15 DF,  p-value: 4.639e-05 |
|  |
| **[1] "Age group Age 80-89"** |
|  |
| **Call:** |
| **lm(formula=Diagnoses ~ Year, data=df)** |
|  |
| Residuals: |
| Min       1Q   Median       3Q      Max |
| -201.902  -74.039    8.647   48.392  226.843 |
|  |
| **Coefficients:** |
| **Estimate Std. Error t value Pr(>\|t\|)** |
| **(Intercept) -2.936e+05  1.053e+04  -27.88 2.45e-14 ***** |
| **Year         1.467e+02  5.232e+00   28.04 2.26e-14 ***** |
| **---** |
| **Signif. codes:  0 ‘***’ 0.001 ‘**’ 0.01 ‘*’ 0.05 ‘.’ 0.1 ‘ ’ 1** |
|  |
| Residual standard error: 105.7 on 15 degrees of freedom |
| Multiple R-squared:  0.9813, |
| F-statistic: 786.1 on 1 and 15 DF,  p-value: 2.256e-14 |
|  |
| **"Age group Age 90+"** |
|  |
| **Call:** |
| **lm(formula=Diagnoses ~ Year, data=df)** |
|  |
| Residuals: |
| Min       1Q   Median       3Q      Max |
| -21.3333  -9.6961   0.0343   9.0294  22.3922 |
|  |
| **Coefficients:** |
| **Estimate Std. Error t value Pr(>\|t\|)** |
| **(Intercept) -32447.044   1354.654  -23.95 2.28e-13 ***** |
| **Year            16.181      0.673   24.05 2.15e-13 ***** |
| **---** |
| **Signif. codes:  0 ‘***’ 0.001 ‘**’ 0.01 ‘*’ 0.05 ‘.’ 0.1 ‘ ’ 1** |
|  |
| Residual standard error: 13.59 on 15 degrees of freedom |
| Multiple R-squared:  0.9747, |
| F-statistic: 578.2 on 1 and 15 DF,  p-value: 2.154e-13 |
|  |
| **"Age group Total"** |
|  |
| **Call:** |
| **lm(formula=Diagnoses ~ Year, data=df)** |
|  |
| Residuals: |
| Min      1Q  Median      3Q     Max |
| -1378.9  -136.9   216.7   263.4   689.2 |
|  |
| **Coefficients:** |
| **Estimate Std. Error t value Pr(>\|t\|)** |
| **(Intercept) -692326.18   52237.10  -13.25 1.10e-09 ***** |
| **Year            347.78      25.95   13.40 9.41e-10 ***** |
| --- |
| Signif. codes:  0 ‘***’ 0.001 ‘**’ 0.01 ‘*’ 0.05 ‘.’ 0.1 ‘ ’ 1 |
|  |
| Residual standard error: 524.2 on 15 degrees of freedom |

This table shows the results of linear regression analyses assessing the relationship between the number of diagnoses of spondylodiscitis (ICD-10: M46.4 + M46.3+ M46.2 between 2005 and 20201) and year, stratified by age groups. The table reports the coefficient estimates, standard errors, t-values, and p-values for the intercept and slope of the regression line for each age group. The table includes nine rows, each representing a different age group, ranging from 0-19 to 90 and above, and a total row for all age groups combined.

**Supplementary Table S4:** Pairwise comparisons using t-tests with pooled SD for number of diagnoses of spondylodiscitis (ICD-10: M46.4 + M46.3+ M46.2, 2005 to 2021) between different age groups.

| **Pairwise comparisons using t tests with pooled SD** | | | | | | | | | |
| --- | --- | --- | --- | --- | --- | --- | --- | --- | --- |
| **data:  Diagnoses  ~ Age** | | | | | | | | | |
|  | **0-19** | **20-29** | **30-39** | **40-49** | **50-59** | **60-69** | **70-79** | **80-89** | **90+** |
| **Age 20-29** | 1.00000 | - | - | - | - | - | - | - | - |
| **Age 30-39** | 1.00000 | 1.00000 | - | - | - | - | - | - | - |
| **Age 40-49** | 1.00000 | 1.00000 | 1.00000 | - | - | - | - | - | - |
| **Age 50-59** | 0.0050 | 0.0040 | 0.0260 | 0.8707 | - | - | - | - | - |
| **Age 60-69** | 2.7e-09 | 1.9e-09 | 3.0e-08 | 9.6e-06 | 0.1168 | - | - | - | - |
| **Age 70-79** | < 2e-16 | < 2e-16 | < 2e-16 | 4.4e-16 | 5.1e-10 | 0.0016 | - | - | - |
| **Age 80-89** | 4.4e-09 | 3.2e-09 | 4.8e-08 | 1.5e-05 | 0.1554 | 1.0000 | 0.0011 | - | - |
| **Age 90+** | 1.00000 | 1.00000 | 1.00000 | 1.00000 | 0.0135 | 1.1e-08 | < 2e-16 | 1.8e-08 | - |
| **Total** | < 2e-16 | < 2e-16 | < 2e-16 | < 2e-16 | <  2e-16 | < 2e-16 | < 2e-16 | < 2e-16 | < 2e-16 |
| **P value adjustment method: Bonferroni** | | | | | | | | | |

This table presents the results of pairwise comparisons using t-tests with pooled standard deviation for the number of diagnoses of spondylodiscitis (ICD-10: M46.4 + M46.3+ M46.2, 2005 to 2021) between different age groups. Each row and column of the table represents an age group, ranging from 0-19 to 90 and above. Each cell of the table represents the P value for the comparison of two age groups. The p-values for all the pairwise comparisons were adjusted for multiple comparisons using the Bonferroni correction method to control for the possibility of type I errors.

**Supplementary Table S5:** Univariate linear regression analysis of the relationship between the number of deaths in the context of spondylodiscitis (ICD-10: M46.4 + M46.3+ M46.2, 2005 to 2021) and various factors.

| **"Age group Age 0-19"** |
| --- |
|  |
| **Call:** |
| **lm(formula=Death ~ Year, data=df)** |
|  |
| Residuals: |
| Min     1Q Median     3Q    Max |
| 0      0      0      0      0 |
|  |
| **Coefficients:** |
| **Estimate Std. Error t value Pr(>\|t\|)** |
| **(Intercept)        0          0     NaN      NaN** |
| **Year               0          0     NaN      NaN** |
|  |
| Residual standard error: 0 on 15 degrees of freedom |
| Multiple R-squared:    NaN, Adjusted R-squared:    NaN |
| F-statistic:   NaN on 1 and 15 DF,  p-value: NA |
|  |
| **"Age group Age 20-29"** |
|  |
| **Call:** |
| **lm(formula=Death ~ Year, data=df)** |
|  |
| Residuals: |
| Min       1Q   Median       3Q      Max |
| -0.17647 -0.10294 -0.04412  0.01471  0.85294 |
|  |
| **Coefficients:** |
| **Estimate Std. Error t value Pr(>\|t\|)** |
| **(Intercept) -29.54412   23.76451  -1.243    0.233** |
| **Year          0.01471    0.01181   1.246    0.232** |
|  |
| Residual standard error: 0.2385 on 15 degrees of freedom |
| Multiple R-squared:  0.09375, Adjusted R-squared:  0.03333 |
| F-statistic: 1.552 on 1 and 15 DF,  p-value: 0.232 |
|  |
| **"Age group Age 30-39"** |
|  |
| **Call:** |
| **lm(formula=Death ~ Year, data=df)** |
|  |
| Residuals: |
| Min      1Q  Median      3Q     Max |
| -0.3529 -0.3529 -0.3529  0.6471  0.6471 |
|  |
| **Coefficients:** |
| **Estimate Std. Error t value Pr(>\|t\|)** |
| **(Intercept) 3.529e-01  5.070e+01   0.007    0.995** |
| **Year        1.206e-15  2.519e-02   0.000    1.000** |
|  |
| Residual standard error: 0.5087 on 15 degrees of freedom |
| Multiple R-squared:  1.521e-28, Adjusted R-squared:  -0.06667 |
| F-statistic: 2.282e-27 on 1 and 15 DF,  p-value: 1 |
|  |
| **"Age group Age 40-49"** |
|  |
| **Call:** |
| **lm(formula=Death ~ Year, data=df)** |
|  |
| Residuals: |
| Min      1Q  Median      3Q     Max |
| -2.7279 -1.1618 -0.0049  1.1275  2.9828 |
|  |
| **Coefficients:** |
| **Estimate Std. Error t value Pr(>\|t\|)** |
| **(Intercept) -287.80147  152.22576  -1.891   0.0782 .** |
| **Year           0.14461    0.07562   1.912   0.0751 .** |
| **---** |
| **Signif. codes:  0 ‘***’ 0.001 ‘**’ 0.01 ‘*’ 0.05 ‘.’ 0.1 ‘ ’ 1** |
|  |
| Residual standard error: 1.527 on 15 degrees of freedom |
| Multiple R-squared:  0.196, Adjusted R-squared:  0.1424 |
| F-statistic: 3.657 on 1 and 15 DF,  p-value: 0.07513 |
|  |
| **"Age group Age 50-59"** |
|  |
| **Call:** |
| **lm(formula=Death ~ Year, data=df)** |
|  |
| Residuals: |
| Min      1Q  Median      3Q     Max |
| -7.0441 -2.2990 -0.2353  1.9240  7.8284 |
|  |
| **Coefficients:** |
| **Estimate Std. Error t value Pr(>\|t\|)** |
| **(Intercept) -1931.6250   395.8382  -4.880 0.000200 ***** |
| **Year            0.9681     0.1966   4.923 0.000184 ***** |
| **---** |
| **Signif. codes:  0 ‘***’ 0.001 ‘**’ 0.01 ‘*’ 0.05 ‘.’ 0.1 ‘ ’ 1** |
|  |
| Residual standard error: 3.972 on 15 degrees of freedom |
| Multiple R-squared:  0.6177, Adjusted R-squared:  0.5923 |
| F-statistic: 24.24 on 1 and 15 DF,  p-value: 0.0001838 |
|  |
| **[1] "Age group Age 60-69"** |
|  |
| **Call:** |
| **lm(formula=Death ~ Year, data=df)** |
|  |
| Residuals: |
| Min       1Q   Median       3Q      Max |
| -14.2353  -3.0441   0.3676   3.1471  14.5735 |
|  |
| **Coefficients:** |
| **Estimate Std. Error t value Pr(>\|t\|)** |
| **(Intercept) -5185.4853   745.2154  -6.958 4.59e-06 ***** |
| **Year            2.6029     0.3702   7.031 4.06e-06 ***** |
| **---** |
| **Signif. codes:  0 ‘***’ 0.001 ‘**’ 0.01 ‘*’ 0.05 ‘.’ 0.1 ‘ ’ 1** |
|  |
| Residual standard error: 7.478 on 15 degrees of freedom |
| Multiple R-squared:  0.7672, Adjusted R-squared:  0.7517 |
| F-statistic: 49.44 on 1 and 15 DF,  p-value: 4.063e-06 |
|  |
| **"Age group Age 70-79"** |
|  |
| **Call:** |
| **lm(formula=Death ~ Year, data=df)** |
|  |
| Residuals: |
| Min      1Q  Median      3Q     Max |
| -20.961 -11.172  -2.635  14.471  32.755 |
|  |
| **Coefficients:** |
| **Estimate Std. Error t value Pr(>\|t\|)** |
| **(Intercept) -1.833e+04  1.733e+03  -10.58 2.37e-08 ***** |
| **Year         9.179e+00  8.608e-01   10.66 2.13e-08 ***** |
| **---** |
| **Signif. codes:  0 ‘***’ 0.001 ‘**’ 0.01 ‘*’ 0.05 ‘.’ 0.1 ‘ ’ 1** |
|  |
| Residual standard error: 17.39 on 15 degrees of freedom |
| Multiple R-squared:  0.8834, Adjusted R-squared:  0.8757 |
| F-statistic: 113.7 on 1 and 15 DF,  p-value: 2.134e-08 |
|  |
| **"Age group Age 80-89"** |
|  |
| **Call:** |
| **lm(formula=Death ~ Year, data=df)** |
|  |
| Residuals: |
| Min      1Q  Median      3Q     Max |
| -40.645  -7.245   1.836  10.076  25.716 |
|  |
| **Coefficients:** |
| **Estimate Std. Error t value Pr(>\|t\|)** |
| **(Intercept) -3.583e+04  1.764e+03  -20.31 2.52e-12 ***** |
| **Year         1.788e+01  8.763e-01   20.41 2.36e-12 ***** |
| **---** |
| **Signif. codes:  0 ‘***’ 0.001 ‘**’ 0.01 ‘*’ 0.05 ‘.’ 0.1 ‘ ’ 1** |
|  |
| Residual standard error: 17.7 on 15 degrees of freedom |
| Multiple R-squared:  0.9652, Adjusted R-squared:  0.9629 |
| F-statistic: 416.4 on 1 and 15 DF,  p-value: 2.36e-12 |
|  |
| **"Age group Age 90+"** |
|  |
| **Call:** |
| **lm(formula=Death ~ Year, data=df)** |
|  |
| Residuals: |
| Min      1Q  Median      3Q     Max |
| -6.2353 -3.1176  0.4118  3.4706  7.0588 |
|  |
| **Coefficients:** |
| **Estimate Std. Error t value Pr(>\|t\|)** |
| **(Intercept) -5781.2941   415.0059  -13.93 5.49e-10 ***** |
| **Year            2.8824     0.2062   13.98 5.22e-10 ***** |
| **---** |
| **Signif. codes:  0 ‘***’ 0.001 ‘**’ 0.01 ‘*’ 0.05 ‘.’ 0.1 ‘ ’ 1** |
|  |
| Residual standard error: 4.164 on 15 degrees of freedom |
| Multiple R-squared:  0.9287, Adjusted R-squared:  0.924 |
| F-statistic: 195.5 on 1 and 15 DF,  p-value: 5.219e-10 |
|  |
| **"Age group Total"** |
|  |
| **Call:** |
| **lm(formula=Death ~ Year, data=df)** |
|  |
| Residuals: |
| Min      1Q  Median      3Q     Max |
| -54.681 -18.667   6.289  12.662  58.961 |
|  |
| **Coefficients:** |
| **Estimate Std. Error t value Pr(>\|t\|)** |
| **(Intercept) -67378.515   2618.814  -25.73 7.99e-14 ***** |
| **Year            33.672      1.301   25.88 7.32e-14 ***** |
| **---** |
| **Signif. codes:  0 ‘***’ 0.001 ‘**’ 0.01 ‘*’ 0.05 ‘.’ 0.1 ‘ ’ 1** |
|  |
| Residual standard error: 26.28 on 15 degrees of freedom |
| Multiple R-squared:  0.9781, Adjusted R-squared:  0.9766 |
| F-statistic: 669.9 on 1 and 15 DF,  p-value: 7.318e-14 |

This table shows the results of linear regression analyses assessing the relationship between the number of deaths after in-patient stay for spondylodiscitis (ICD-10: M46.4 + M46.3+ M46.2 between 2005 and 20201) and year, stratified by age groups. The table reports the coefficient estimates, standard errors, t-values, and p-values for the intercept and slope of the regression line for each age group. The table includes nine rows, each representing a different age group, ranging from 0-19 to 90 and above, and a total row for all age groups combined.

**Supplementary Table S6:** Pairwise comparisons using t-tests with pooled SD for number of deaths in the context of spondylodiscitis (ICD-10: M46.4 + M46.3+ M46.2, 2005 to 2021) between different age groups.

| **Pairwise comparisons using t tests with pooled SD** | | | | | | | | | |
| --- | --- | --- | --- | --- | --- | --- | --- | --- | --- |
| **data:  Death  ~ Age** | | | | | | | | | |
|  | **0-19** | **20-29** | **30-39** | **40-49** | **50-59** | **60-69** | **70-79** | **80-89** | **90+** |
| **Age 20-29** | 1.00000 | - | - | - | - | - | - | - | - |
| **Age 30-39** | 1.00000 | 1.00000 | - | - | - | - | - | - | - |
| **Age 40-49** | 1.00000 | 1.00000 | 1.00000 | - | - | - | - | - | - |
| **Age 50-59** | 1.00000 | 1.00000 | 1.00000 | 1.00000 | - | - | - | - | - |
| **Age 60-69** | 0.65257 | 0.65730 | 0.68137 | 0.96869 | 1.00000 | - | - | - | - |
| **Age 70-79** | 1.7e-08 | 1.7e-08 | 1.9e-08 | 3.8e-08 | 9.8e-07 | 0.00194 | - | - | - |
| **Age 80-89** | 6.4e-10 | 6.5e-10 | 7.0e-10 | 1.5e-09 | 4.5e-08 | 0.00016 | 1.00000 | - | - |
| **Age 90+** | 1.00000 | 1.00000 | 1.00000 | 1.00000 | 1.00000 | 1.00000 | 2.2e-06 | 1.1e-07 | - |
| **Total** | < 2e-16 | < 2e-16 | < 2e-16 | < 2e-16 | <  2e-16 | < 2e-16 | < 2e-16 | < 2e-16 | < 2e-16 |
| **P value adjustment method: Bonferroni** | | | | | | | | | |

This table presents the results of pairwise comparisons using t-tests with pooled standard deviation for the number of deaths after in-patient stay for spondylodiscitis (ICD-10: M46.4 + M46.3+ M46.2, 2005 to 2021) between different age groups. Each row and column of the table represents an age group, ranging from 0-19 to 90 and above. Each cell of the table represents the P value for the comparison of two age groups. The p-values for all the pairwise comparisons were adjusted for multiple comparisons using the Bonferroni correction method to control for the possibility of type I errors.

**Supplementary Table S7:** Multivariate linear regression analysis of the relationship between the number of deaths in the context of spondylodiscitis (ICD-10: M46.4 + M46.3+ M46.2, 2005 to 2021) and various explanatory variables.

| **Linear regression** | | | | |
| --- | --- | --- | --- | --- |
| **Call: lm(formula=Death ~ as.factor(Year) + as.factor(Age) + Diagnoses +**  **Incidence + LOS + `Regular DC` + `DC against medical advise` +**  **`Transfer to other hospital` + `DC to rehab/care home/hospice` +**  **`Proportion of DC being Death` + `Death:Diagnoses` + `Diagnoses w/spinal fusion` +**  **`Diagnoses w/vertebral body replacement` + `Diagnoses w/complex spinal reconstruction`,**  **data=GermanSpondy)** | | | | |
|  | Estimate. | Std. Error. | t value | Pr(>\|t\|) |
| **(Intercept)** | -2.721282 | 4.208274 | -0.647 | 0.519973 |
| **as.factor(Year)2006** | 2.310629 | 1.854064 | 1.246 | 0.216827 |
| **as.factor(Year)2007** | 1.978837 | 1.920139 | 1.031 | 0.306290 |
| **as.factor(Year)2008** | 3.368293 | 2.074898 | 1.623 | 0.109009 |
| **as.factor(Year)2009** | 1.319985 | 2.010261 | 0.657 | 0.513576 |
| **as.factor(Year)2010** | 0.474864 | 2.338553 | 0.203 | 0.839678 |
| **as.factor(Year)2011** | -2.131966 | 2.344477 | -0.909 | 0.366282 |
| **as.factor(Year)2012** | 0.674119 | 2..211570 | 0.305 | 0.761412 |
| **as.factor(Year)2013** | 3.274009 | 2.070152 | 1.582 | 0.118265 |
| **as.factor(Year)2014** | 1.524735 | 2.204334 | 0.692 | 0.491414 |
| **as.factor(Year)2015** | 2.805301 | 2.316976 | 1.211 | 0.230060 |
| **as.factor(Year)2016** | 3.016230 | 2.200058 | 1.371 | 0.174761 |
| **as.factor(Year)2017** | 4.479895 | 2.271017 | 1.973 | 0.052488 . |
| **as.factor(Year)2018** | 1.362773 | 2.081612 | 0.655 | 0.514824 |
| **as.factor(Year)2019** | -1.568876 | 2.148828 | -0.730 | 0.467761 |
| **as.factor(Year)2020** | -0.307736 | 2.156181 | -0.143 | 0.886919 |
| **as.factor(Year)2021** | 1.445205 | 2.249057 | 0.643 | 0.522594 |
| **as.factor(Age)Age 20-29** | -1.952062 | 3.965149 | -0.492 | 0.624044 |
| **as.factor(Age)Age 30-39** | -3.737807 | 3.564047 | -1.049 | 0.297899 |
| **as.factor(Age)Age 40-49** | -6.926323 | 3.776103 | -1.834 | 0.070867 . |
| **as.factor(Age)Age 50-59** | -11.499776 | 4.386538 | -2.622 | 0.010729 * |
| **as.factor(Age)Age 60-69** | -16.422385 | 5.142301 | -3.194 | 0.002107 ** |
| **as.factor(Age)Age 70-79** | -18.551794 | 5.776690 | -3.211 | 0.001996 ** |
| **as.factor(Age)Age 80-89** | -9.193826 | 5.659014 | -1.625 | 0.108735 |
| **as.factor(Age)Age 90+** | -7.051267 | 7.878472 | -0.895 | 0.373852 |
| **as.factor(Age)Total** | -61.970346 | 9.036242 | -6.858 | 2.26e-09 *** |
| **Diagnoses** | 0.908811 | 0.025426 | 35.743 | < 2e-16 *** |
| **Incidence** | -27.242281 | 23.494728 | -1.160 | 0.250190 |
| **LOS** | 0.039931 | 0.161766 | 0.247 | 0.805749 |
| **`Regular DC`** | -0.864386 | 0.023104 | 37.412 | < 2e-16 *** |
| **`DC against medical advise`** | -0.628065 | 0.080642 | -7.788 | 4.48e-11 *** |
| **`Transfer to other hospital`** | -0.879171 | 0.031503 | -27.907 | < 2e-16 *** |
| **`DC to rehab/care home/hospice`** | -0.836478 | 0.034592 | -24.181 | < 2e-16 *** |
| **`Proportion of DC being Death`** | -16.152031 | 14.812974 | -1.090 | 0.279277 |
| **`Death:Diagnoses`** | 64.193723 | 45.408804 | 1.414 | 0.161886 |
| **`Diagnoses w/spinal fusion`** | 0.012896 | 0.003375 | 3.821 | 0.000284 *** |
| **`Diagnoses w/vertebral body replacement`** | - 0.089064 | 0.028466 | -3.129 | 0.002559 ** |
| **`Diagnoses w/complex spinal reconstruction`** | 0.044205 | 0.085309 | 0.518 | 0.605972 |
| **---** | | | | |
| **Signif. codes:  0 ‘***’ 0.001 ‘**’ 0.01 ‘*’ 0.05 ‘.’ 0.1 ‘ ’ 1** | | | | |
|  | | | | |
| **Residual standard error: 3.017 on 70 degrees of freedom** | | | | |
| **(62 observations deleted due to missingness)** | | | | |
| **Multiple R-squared:  0.9998,** **Adjusted R-squared:  0.9996** | | | | |
| **F-statistic:  7796 on 37 and 70 DF,  p-value:<2.2e-16** | | | | |

This table presents the results of a linear regression analysis of the relationship between the number of deaths after in-patient stay for spondylodiscitis (ICD-10: M46.4 + M46.3+ M46.2, 2005 to 2021) and various factors including year, age, incidence, length of stay (LOS), types of discharge, death, transfer to other hospital, proportion of discharge being death, diagnoses with spinal fusion, vertebral body replacement, and complex spinal reconstruction. The table provides the coefficient estimates, standard errors, t-values, and p-values for each factor. The results are presented for each year from 2006 to 2021 (2005 is the intercept) and for each age group ranging from 20-29 to 90 and above, as well as a total row for all age groups combined. The table highlights statistically significant values using asterisks, where *** denotes a p-value less than 0.001, ** denotes a p-value less than 0.01, and * denotes a p-valueless than 0.05. The table also provides information on the residual standard error, multiple R-squared, adjusted R-squared, and F-statistic for the overall regression model. The analysis involved 37 degrees of freedom and excluded 62 observations due to missing data.

**Supplementary Table S8:** Multivariate linear regression analysis of the relationship between the length of stay for spondylodiscitis (ICD-10: M46.4 + M46.3+ M46.2, 2005 to 2021) and various explanatory variables.

| **Linear regression** | | | | |
| --- | --- | --- | --- | --- |
| **Call: lm(formula= LOS ~ 0 + as. factor(Year) + as. factor(Age) + Incidence +**  **Death + 'Regular DC' + 'DC against medical advise’ + Diagnoses +**  **`Transfer to other hospital` + `DC to rehab/care home/hospice` +**  **`Proportion of DC being Death` + `Death:Diagnoses` + `Diagnoses w/spinal fusion` +**  **`Diagnoses w/vertebral body replacement` + `Diagnoses w/complex spinal reconstruction`,**  **Data = GermanSpondy)** | | | | |
|  | Estimate. | Std. Error. | t value | Pr(>\|t\|) |
| **(as.factor(Year)2005** | 14.17 | 2.520 | 5.626 | 3.54E-07 *** |
| **as.factor(Year)2006** | 13.66 | 2.460 | 5.551 | 4.76E-07 *** |
| **as.factor(Year)2007** | 12.26 | 2.490 | 4.904 | 5.87E-06 *** |
| **as.factor(Year)2008** | 14.97 | 2.510 | 5.965 | 9.04E-08 *** |
| **as.factor(Year)2009** | 13.41 | 2.090 | 6.403 | 1.50E-08 *** |
| **as.factor(Year)2010** | 12.54 | 2.576 | 4.868 | 6.73E-06 *** |
| **as.factor(Year)2011** | 11.66 | 2.642 | 4.415 | 3.60E-05 *** |
| **as.factor(Year)2012** | 12.69 | 2.598 | 4.884 | 6.34E-06 *** |
| **as.factor(Year)2013** | 10.90 | 2.576 | 4.231 | 6.94E-05 *** |
| **as.factor(Year)2014** | 11.18 | 2.657 | 4.207 | 7.55E-05 *** |
| **as.factor(Year)2015** | 7.03 | 2.689 | 2.616 | 0.010902 * |
| **as.factor(Year)2016** | 6.73 | 2.571 | 2.619 | 0.010803 * |
| **as.factor(Year)2017** | 7.51 | 2.531 | 2.968 | 0.004102 ** |
| **as.factor(Year)2018** | 8.42 | 2.548 | 3.307 | 0.001492 ** |
| **as.factor(Year)2019** | 8.10 | 2.632 | 3.076 | 0.002991 ** |
| **as.factor(Year)2020** | 6.56 | 2.673 | 2.454 | 0.016628 * |
| **as.factor(Year)2021** | 8.44 | 2.615 | 3.226 | 0.001907 ** |
| **as.factor(Age)Age 20-29** | 6.23 | 2.705 | 2.304 | 0.024209 * |
| **as.factor(Age)Age 30-39** | 5.60 | 2.394 | 2.340 | 0.022127 * |
| **as.factor(Age)Age 40-49** | 6.65 | 2.550 | 2.609 | 0.011103* |
| **as.factor(Age)Age 50-59** | 10.62 | 2.917 | 3.639 | 0.000519 *** |
| **as.factor(Age)Age 60-69** | 14.08 | 3.421 | 4.114 | 0.000104 *** |
| **as.factor(Age)Age 70-79** | 16.85 | 3.742 | 4.503 | 2.61E-05 *** |
| **as.factor(Age)Age 80-89** | 15.72 | 3.490 | 4.504 | 2.60E-05 *** |
| **as.factor(Age)Age 90+** | 20.73 | 5.107 | 4.060 | 0.000126 *** |
| **as.factor(Age)Total** | 19.16 | 7.469 | 2.565 | 0.012464 * |
| **Incidence** | 0.25 | 0.078 | 3.210 | 0.002006 ** |
| **Death** | 0.06 | 0.082 | 0.703 | 0.484312 |
| **`Regular DC`** | 0.05 | 0.074 | 0.665 | 0.508439 |
| **`DC against medical advise`** | 0.09 | 0.080 | 1.115 | 0.268623 |
| **Diagnoses** | -0.04 | 0.075 | -0.593 | 0.555282 |
| **`Transfer to other hospital`** | 0.03 | 0.076 | 0.374 | 0.709697 |
| **`DC to rehab/care home/hospice`** | 0.04 | 0.073 | 0.545 | 0.587518 |
| **`Proportion of DC being Death`** | 17.22 | 10.160 | 1.695 | 0.094427 |
| **`Death:Diagnoses`** | -97.90 | 32.580 | -3.005 | 0.003680 ** |
| **`Diagnoses w/spinal fusion`** | 0.00 | 0.002 | 0.136 | 0.891841 |
| **`Diagnoses w/vertebral body replacement`** | -0.01 | 0.020 | -0.482 | 0.631161 |
| **`Diagnoses w/complex spinal reconstruction`** | 0.03 | 0.054 | 0.486 | 0.628143 |
| **---** | | | | |
| **Signif. codes:  0 ‘***’ 0.001 ‘**’ 0.01 ‘*’ 0.05 ‘.’ 0.1 ‘ ’ 1** | | | | |
|  | | | | |
| **Residual standard error: 2.083 on 70 degrees of freedom** | | | | |
| **(62 observations deleted due to missingness)** | | | | |
| **Multiple R-squared:  0.995,** **Adjusted R-squared:  0.9923** | | | | |
| **F-statistic:  368.5 on 38 and 70 DF,  p-value:<2.2e-16** | | | | |

This table displays results from a linear regression analysis examining the relationship between the length of stay post in-patient treatment for spondylodiscitis, using ICD-10 codes M46.4, M46.3, and M46.2, from the years 2005 to 2021. The regression considers various factors: Year of Admission (2006-2021, with 2005 as the reference or intercept), Age Groups (ranging from 20-29 up to 90+), Incidence Rate (the frequency of new cases during a specified time), Death Rate (the proportion of discharges resulting in death), Types of Discharges (including regular discharge, discharge against medical advice, transfers to other hospitals, and transfers to rehabilitation/care homes/hospice), and Diagnoses & Interventions (diagnoses associated with spinal fusion, vertebral body replacement, and complex spinal reconstruction). Metrics provided for each factor include the coefficient estimates, standard errors, t-values, and p-values. Significance is indicated with asterisks: '' for p-values < 0.001, '' for p-values < 0.01, and '' for p-values < 0.05. Additional metrics such as the residual standard error, multiple R-squared, adjusted R-squared, and F-statistic offer insights into the model's fit and reliability. The analysis is based on 45 degrees of freedom and excludes 62 observations due to missingness.
